# Space microgravity increases expression of genes associated with proliferation and differentiation in human cardiac spheres

**DOI:** 10.1038/s41526-023-00336-6

**Published:** 2023-12-09

**Authors:** Hyun Hwang, Antonio Rampoldi, Parvin Forghani, Dong Li, Jordan Fite, Gene Boland, Kevin Maher, Chunhui Xu

**Affiliations:** 1 Department of Pediatrics, Emory University School of Medicine and Children’s Healthcare of Atlanta, Atlanta, GA USA; 2 Techshot, Inc., Greenville, IN USA; 3 Wallace H. Coulter Department of Biomedical Engineering, Georgia Institute of Technology and Emory University, Atlanta, GA USA

**Keywords:** Cell biology, Molecular medicine

## Abstract

Efficient generation of cardiomyocytes from human-induced pluripotent stem cells (hiPSCs) is important for their application in basic and translational studies. Space microgravity can significantly change cell activities and function. Previously, we reported upregulation of genes associated with cardiac proliferation in cardiac progenitors derived from hiPSCs that were exposed to space microgravity for 3 days. Here we investigated the effect of long-term exposure of hiPSC-cardiac progenitors to space microgravity on global gene expression. Cryopreserved 3D hiPSC-cardiac progenitors were sent to the International Space Station (ISS) and cultured for 3 weeks under ISS microgravity and ISS 1 G conditions. RNA-sequencing analyses revealed upregulation of genes associated with cardiac differentiation, proliferation, and cardiac structure/function and downregulation of genes associated with extracellular matrix regulation in the ISS microgravity cultures compared with the ISS 1 G cultures. Gene ontology analysis and Kyoto Encyclopedia of Genes and Genomes mapping identified the upregulation of biological processes, molecular function, cellular components, and pathways associated with cell cycle, cardiac differentiation, and cardiac function. Taking together, these results suggest that space microgravity has a beneficial effect on the differentiation and growth of cardiac progenitors.

## Introduction

Human-induced pluripotent stem cells (hiPSCs) may regenerate indefinitely and undergo differentiation into any cell type depending on the protocols used. In this aspect, understanding how cardiomyocytes can be obtained and grown from differentiating hiPSCs is of great importance for their applications in disease modeling, drug development, and regenerative medicine^1–3^. Currently, there are a few limitations regarding the use of hiPSC-derived cardiomyocytes (hiPSC-CMs), including a large number of cells needed for cardiac regeneration. Understanding and improving the pathways involved in cardiomyocyte proliferation and differentiation is clearly of great interest given the potential application of these cells. Investigating cardiac progenitor cells, the intermediate stage of the cells derived from hiPSCs could help improve the efficiency of hiPSC-CM generation.

Microgravity can significantly change cell properties such as cell metabolism and function^4–7^. Exposure of cardiac progenitors to microgravity has the potential to serve as a new method to increase cardiomyocyte differentiation efficiency. Simulated microgravity has been used in studies on animal models^8–10^ and cardiomyocytes from animals^11^, but relatively fewer studies focused on the effect of microgravity on cardiac progenitors or stem cell-derived cardiomyocytes^12,13^. We previously discovered that three-dimensional (3D) cardiac progenitors under a simulated microgravity environment created with a random positioning machine generated enriched cardiomyocytes at high cell yield compared with parallel cultures under standard gravity^14^. Microgravity, however, cannot be perfectly replicated on Earth, since gravitational forces are still present under simulated microgravity.

Space experimentation offers a great opportunity to explore the potential impact of microgravity on cells. This is why the International Space Station (ISS) U.S. National Laboratory and other national and space agencies are further increasing experiments and studies on space microgravity regarding biology and disease modeling^15–17^. In a recent spaceflight experiment, we found that a short-term (3 days) exposure of cardiac progenitors derived from hiPSCs to space microgravity increased the expression of genes associated with proliferation^18^. Here, we further investigated the effect of a long-term (3 weeks) exposure of cardiac progenitors to space microgravity on changes in gene expression of late-stage cardiomyocytes.

## Results

### Overall experimental design

**Fig. 1:**
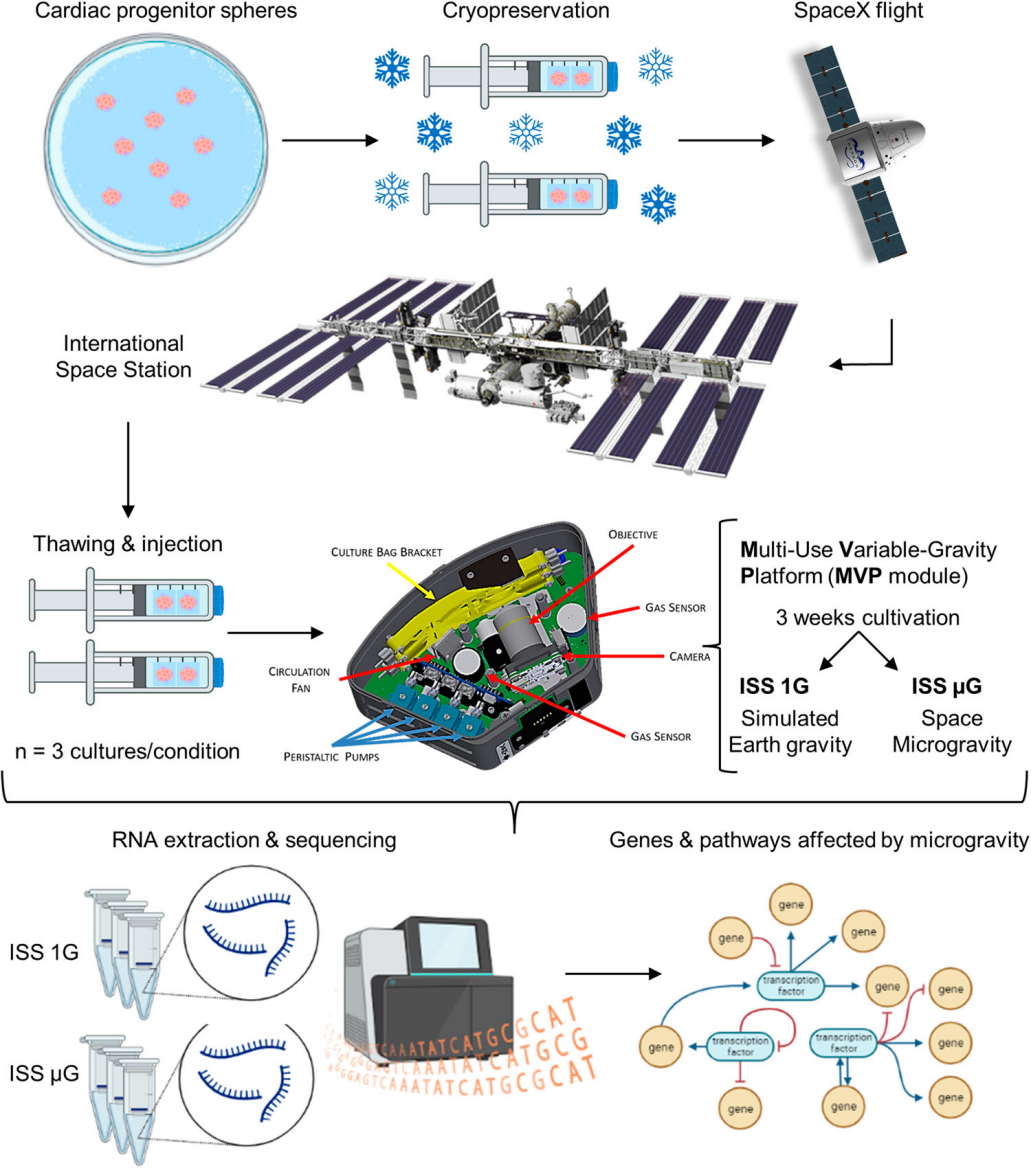
Schematic of spaceflight operation. Schematic of sample preparation, spaceflight operation, multi-specimen variable-gravity platform (MVP) sample collection, RNA extraction, sequencing, and gene expression analyses. Figures created with BioRender. MVP module image source: Techshot Inc, SpaceX-20 Dragon capsule, and International Space Station image source: NicePNG (images adapted by the author).

As shown in the schematic of operational procedure for our spaceflight experiment (Fig. 1), the cryopreserved cardiac progenitor spheres were generated on the ground and sent to the ISS through the SPACEX-20 mission, a mission launched by the aerospace company SpaceX on March 6, 2020 for the delivery of cargo and supplies to the ISS (https://www.issnationallab.org/launches/spacex-crs-20/). On the ISS, the astronauts successfully thawed the cryopreserved cardiac progenitor spheres, cultured the cells using the multi-specimen variable-gravity platform (MVP) from Techshot, Inc. for 22 days, and then sent live cultures back to us. The MVP system has been successfully used in space experiments^19^ and allows the loading of multiple biological samples under both ISS µG and ISS 1G conditions (the 1G condition in the MVP system on the ISS was achieved by centrifugation). In our experiment, each condition was run in triplicates. The use of ISS 1G samples as a control in our data analyses allowed for the examination of the effect of space microgravity on the cells while keeping other variables constant such as the effect of space radiation. At the end of the spaceflight experiment, we received live cardiac spheres^18^ and then isolated RNA from cardiac spheres in both the ISS μG and the ISS 1G conditions for RNA sequencing (RNA-seq) to compare their global gene expression profiles and examine the effect of space microgravity on transcriptome alterations of cardiac spheres.

### Long-term exposure to space microgravity increases expression of genes associated with cell proliferation and cardiac differentiation

**Fig. 2:**
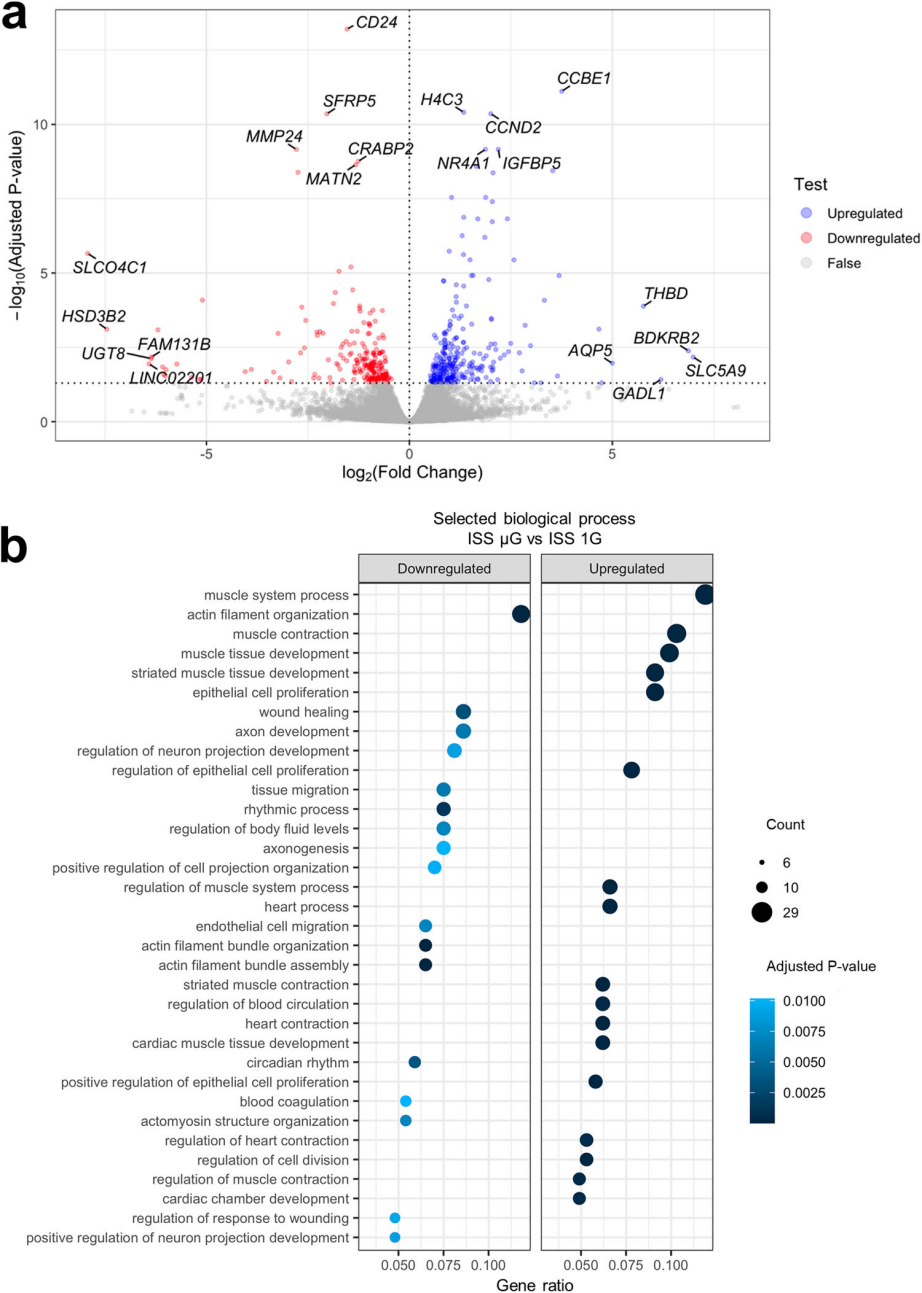
Differentially expressed genes and gene ontology (GO) terms identified by RNA-sequencing analyses of hiPSC-CMs exposed to space microgravity. **a** Volcano plot illustrating differentially expressed genes between ISS µG and ISS 1G samples (*n* = 3 cultures) collected from IMR90 hiPSC differentiation cultures of 3 weeks on the ISS (long-term exposure to space microgravity). **b** Dot plot showing up- and downregulated GO terms of biological processes. GSEA gene set enrichment analysis, ISS 1G the 1G condition on the International Space Station, ISS µG the microgravity condition on the International Space Station.

RNA-seq analyses revealed a total of 470 differentially expressed genes (DEGs) upon applying a threshold of adjusted *p*-value of 0.05 (Fig. 2a). Among the 470 DEGs, 271 were significantly upregulated and 199 were significantly downregulated in the ISS μG samples compared with the ISS 1G samples. The 271 upregulated and 199 downregulated DEGs in terms of log_2_(fold change) value are shown in Tables S1 and S2. The top 10 genes that are noticeable in terms of adjusted *p*-value and log_2_(fold change) are annotated in Fig. 2a.

Among the upregulated DEGs, several were involved in the cell cycle, proliferation, survival, and cardiac differentiation, including *CCBE1*, *CCND2*, *IGFBP5*, and *BDKRB2* (Supplementary Table 1). Collagen and calcium-binding EGF domains 1 (*CCBE1*) was the most significantly upregulated DEG in terms of adjusted *p*-value (log_2_[fold change] 3.75, adjusted *p*-value 7.67E−12). *CCBE1* is important for normal heart development as its downregulation disrupts the differentiation of stem cells into cardiac mesoderm lineage^20^. Cyclin D2 (*CCND2)* was also among the top upregulated DEGs (log_2_[fold change] 2.01, adjusted *p*-value 4.41E−11). *CCND2* is a member of the cyclin family required for progression through the G1 phase of the cell cycle. Insulin-like growth factor binding protein 5 (*IGFBP5*) was the top 5 upregulated DEGs in terms of the adjusted *p*-value (log_2_[fold change] 2.19, adjusted *p*-value 6.94E−10). *IGFBP5* is a member of the IGF signaling pathway that regulates IGF transport and availability during embryonic development, while the IGF signaling pathway plays a key role during embryonic cardiac development, regulating processes of cell proliferation and survival^21^. *BDKRB2* (bradykinin receptor B2, log_2_[fold change] 6.87, adjusted *p*-value 4.13E−03) encodes a G-protein coupled receptor for bradykinin which stimulates the proliferation of smooth muscle cells^22^ and endothelial cells^23^.

*CD24* (CD24 molecule) was identified as the most significantly downregulated DEGs (log_2_[fold change] −1.54, adjusted *p*-value 6.23E−14, Supplementary Table 2). *CD24* was reported as a marker for human pluripotent stem cells^24^ and its downregulation may indicate enhanced cardiac differentiation in ISS µG cells. In addition, ectodermal-neural cortex 1 (*ENC1*) was among the top downregulated DEGs (log_2_[fold change] −2, adjusted *p*-value 8.25E−03, Supplementary Table 2), suggesting limited neural differentiation in the ISS µG cultures.

**Fig. 3:**
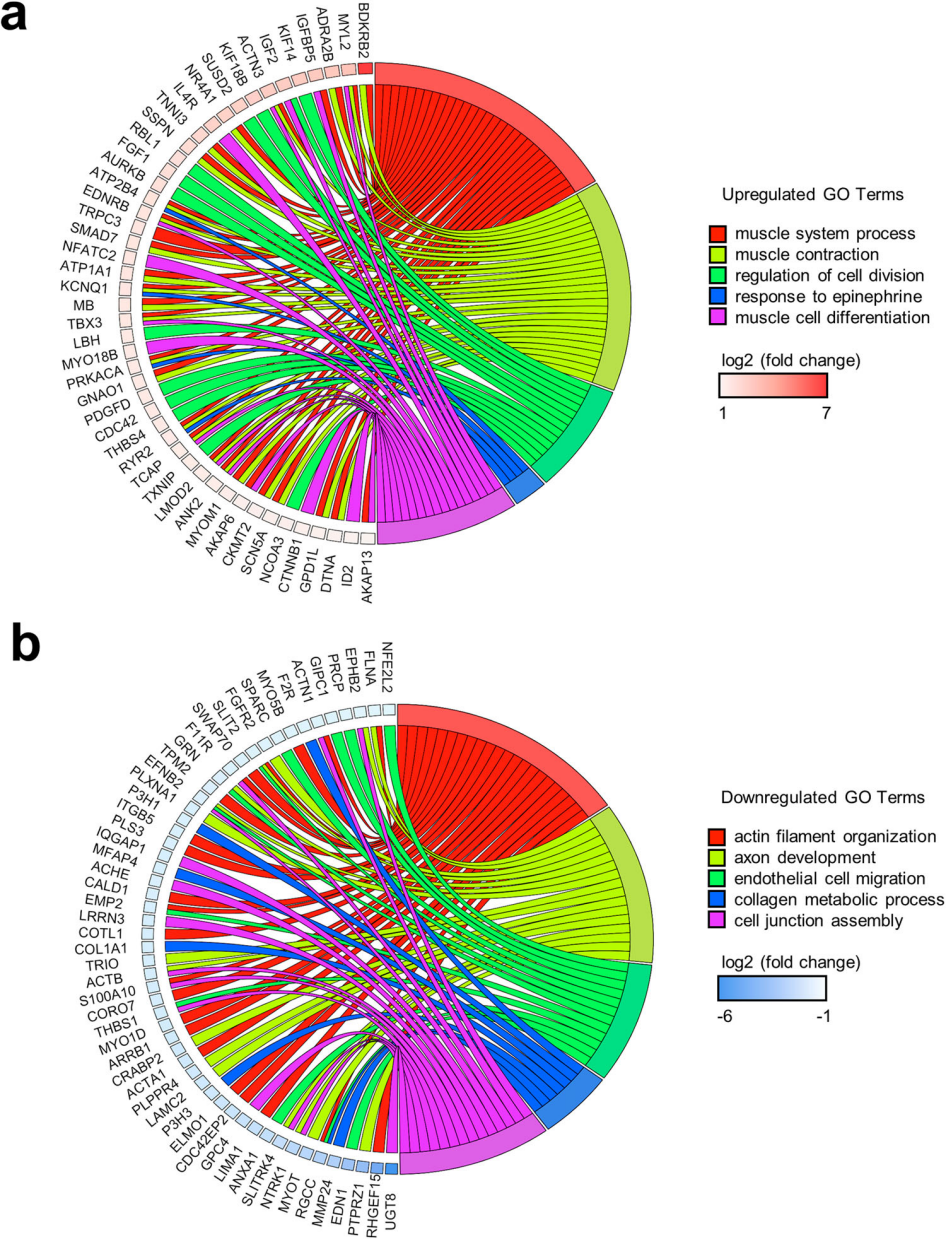
Chord diagrams showing the relationship between gene ontology (GO) terms and differentially expressed genes in hiPSC-CMs exposed to space microgravity. **a** Chord diagram of selected upregulated GO terms and genes in ISS µG vs. ISS 1G of IMR90 hiPSC differentiation cultures of 3 weeks on the ISS. **b** Chord diagram of selected downregulated GO terms and genes. GO terms were presented on the right, genes on the left, and colored squares on the left indicated log_2_(fold change) value from highest to lowest (*n* = 3 cultures).

Consistently, gene ontology (GO) enrichment analysis revealed upregulation of biological processes associated with muscle tissue development (GO:0060537, adjusted *p*-value 7.19E−07), cardiac muscle tissue development (GO:0048738, adjusted *p*-value 1.54E−04), and regulation of cell division (GO:0051302, adjusted *p*-value 1.54E−04) (Fig. 2b, Supplementary Table 3). Upregulated DEGs including *KIF14, IGF2, KIF18B, SUSD2, RBL1, AURKB, LBH, PDGFD, and CDC42* were identified in GO term of regulation of cell division (Fig. 3, Supplementary Table 4). Upregulated DEGs including *MYL2, IGFBP5*, *IGF2, EDNRB*, and *NFATC2* were identified in GO in terms of muscle cell differentiation (Fig. 3, Supplementary Table 5).

Furthermore, the top-downregulated biological processes (Fig. 3, Supplementary Table 6) include axon development (GO:0061564, adjusted *p*-value 6.22E−03), regulation of neuron projection development (GO:0010975, adjusted *p*-value 8.87E−03) and endothelial cell migration (GO:0043542, adjusted *p*-value 7.05E−03), suggesting limited neuronal and endothelial differentiation in the ISS µG cultures, which was consistent with efficient cardiac differentiation.

In addition, Kyoto Encyclopedia of Genes and Genomes (KEGG) pathway mapping showed upregulated genes in the TGF-beta signaling pathway (hsa04350) and adrenergic signaling pathway (hsa04261) (Supplementary Fig. 1). The TGF-beta protein superfamily plays key roles during heart development, and has an important role also in cardiac cell proliferation, differentiation, and heart regeneration^25^. The adrenergic signaling pathway is tightly connected to calcium signaling, cell contraction and can affect cardiomyocyte differentiation and maturation^26^.

### Long-term exposure to space microgravity increases expression of genes associated with cardiac function

Several upregulated genes were involved in increasing cardiac function, including *THBD* (thrombomodulin, log_2_[fold change] 5.76, adjusted *p*-value 1.31E−04), and *GADL1* (glutamate decarboxylase like 1, log_2_[fold change] 6.20, adjusted *p*-value 3.81E−02) (Table S1). Upregulation of *THBD* in hypertrophic cardiomyocytes was shown to decrease cell apoptosis and help maintain a normal contractile function^27^. *GADL1* is involved in the production of carnosine and taurine, while carnosine and taurine are predominantly expressed in muscle tissue, where they regulate calcium signaling and indirectly regulate oxidative stress with their antioxidant effects^28,29^.

GO terms analysis showed that space microgravity upregulated genes associated with biological processes related to muscle system process (GO:0003012, adjusted *p*-value 4.91E−09), muscle contractions (GO:0006936, adjusted *p*-value 7.77E−09), heart process (GO:0003015, adjusted *p*-value 8.21E−05) and heart contraction (GO:0060047, adjusted *p*-value 1.54E−04) (Fig. 2b, Supplementary Table 3). Upregulated DEGs like *MYL2*, *ATP2B4*, *EDNRB*, and *IGF2* were identified in enriched GO terms associated with muscle system process and muscle contraction (Fig. 3).

In addition, upregulation of genes known to be involved with cardiac development and contraction was observed, including *MYL2, TNNI3*, *RYR2, TCAP*, and *SCN5A* (Supplementary Table 7) Genes relevant for cardiac conduction were also observed, including *ATP2B4, PRKACA*, and *ANK2* (Supplementary Table 7).

**Fig. 4:**
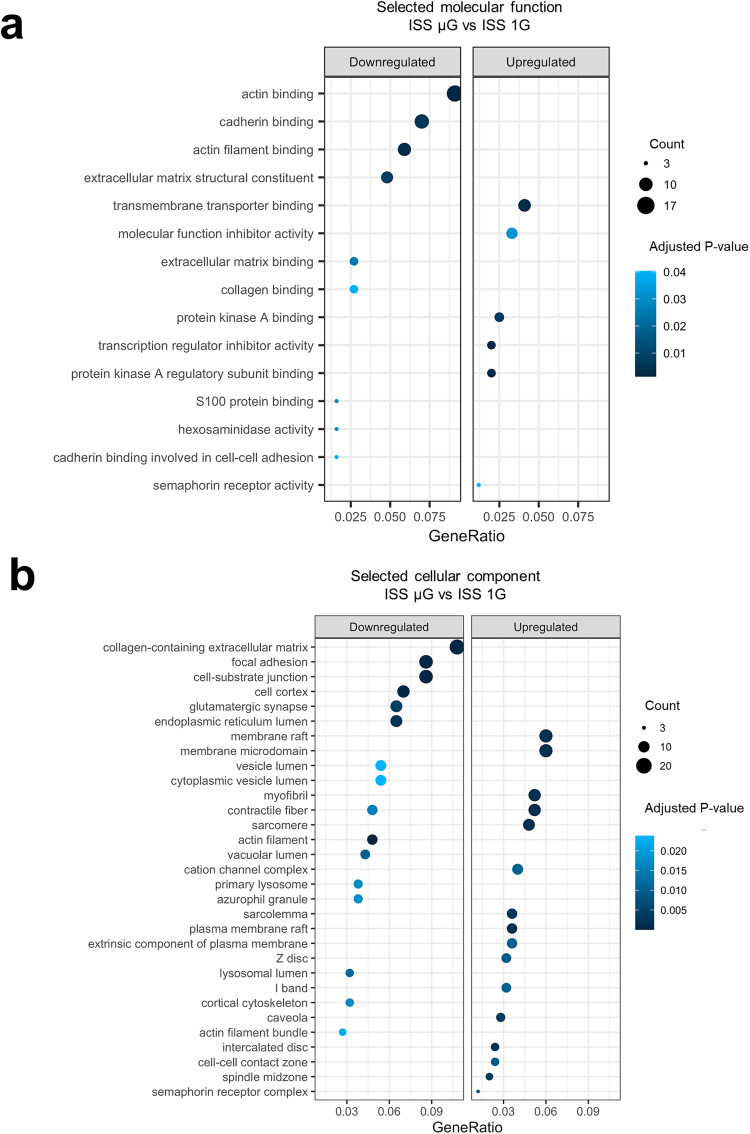
Gene ontology (GO) terms identified by RNA-sequencing analyses of hiPSC-CMs exposed to space microgravity. **a** Dot plot showing upregulated and downregulated genes clustered by GO enrichment analysis of molecular function from ISS µG and ISS 1G samples (*n* = 3 cultures) collected 3 weeks after thawing onboard ISS (long-term exposure to microgravity). **b** Dot plot showing upregulated and downregulated genes clustered by GO enrichment analysis of cellular components. GSEA gene set enrichment analysis, ISS 1G the 1G condition on the International Space Station, ISS µG the microgravity condition on the International Space Station.

Similar results were observed in GO in terms of cellular components and molecular functions associated with upregulated genes involved in muscle structure. These included sarcomere (GO:0030017, adjusted *p*-value 1.76E−03), myofibril (GO:0030016, adjusted *p*-value 1.76E−03), cation channel complex (GO:0034703, adjusted *p*-value 1.04E−02), contractile fiber (GO:0043292, adjusted *p*-value 1.76E−03), z disc (GO:0030018, adjusted *p*-value 9.17E−03) and I band (GO:0031674, adjusted *p*-value 1.05E−02) (Fig. 4, Supplementary Table 3).

GO terms of membrane raft (GO:0045121, adjusted *p*-value 1.86E−03) and membrane microdomain (GO:0098857, adjusted *p*-value 1.86E−03) were among the top upregulated cellular components (Fig. 4, Supplementary Table 3). Membrane/lipid rafts and microdomains play an important role in targeting and controlling the interaction of proteins including cardiac channel subunits localized in lipid rafts^30^.

GO terms related to protein kinase A were also upregulated (Fig. 4, Supplementary Table 3). These included protein kinase A binding (GO:0051018, adjusted *p*-value 7.32E−03) and protein kinase A regulatory subunit binding (GO:0034237, adjusted *p*-value 2.94E−03). Protein kinase A plays a central role in cardiac function by enhancing Ca^2+^ cycling and increasing cardiac muscle contractility^31^. Protein kinase A also regulates fuel supply and energy-generating pathways, moving cardiomyocytes from glucose toward lipid metabolism as an energy source^32^.

### Long-term exposure to space microgravity reduces expression of genes associated with extracellular matrix (ECM) regulation

Our transcriptomics analyses also revealed the downregulation of extracellular structures associated with 3D hiPSC-CMs under space microgravity. For example, downregulated DEGs including *ITIH5*, *COL4A4*, *PTPR21*, COL26A1, and *HPX* were associated with ECM regulation (Supplementary Table 8). Downregulated DEGs including *ITGA11*, *PROCR*, *ANXA1*, and *LIMA1* were associated with focal adhesion (Supplementary Table 9).

GO term analysis performed on downregulated genes revealed downregulation of biological process terms like actin filament organization (GO:0007015, adjusted *p*-value 1.75E−06), wound healing (GO:0042060, adjusted *p*-value 0.00317314), and endothelial cell migration (GO:0043542, adjusted *p*-value 0.00704613) (Fig. 2b, Supplementary Table 6). Downregulated molecular process terms included actin binding (GO:0003779, adjusted *p*-value 1.34E−03), cadherin binding (GO:0045296, adjusted *p*-value 5.84E−03), and collagen binding (GO:0005518, adjusted *p*-value 3.96E−02) (Fig. 4, Supplementary Table 6). Downregulated cellular component terms included collagen-containing extracellular matrix (GO:0062023, adjusted *p*-value 1.45E−06), focal adhesion (GO:0005925, adjusted *p*-value 2.59E−04), primary lysosome (GO:0005766, adjusted *p*-value 1.76E−02) and myofibril (GO:0030016, adjusted *p*-value 3.19E−02) (Fig. 4, Supplementary Table 6). Consistently, KEGG pathway mapping revealed downregulated genes in pathways of ECM–receptor interaction (HSA04512) and focal adhesion (HSA04510) (Supplementary Fig. 2).

### Long-term exposure to space microgravity alters expression of long non-coding RNAs (lncRNAs)

Our transcriptomics analyses revealed altered expression in a limited number of lncRNAs associated with 3D hiPSC-CMs under long-term exposure to space microgravity (Supplementary Tables 1 and 2). Among the 14 lncRNA showing altered expression, 9 were upregulated and 5 downregulated. Two among the 9 upregulated lncRNA were recently found to have important roles in cardiomyocyte development and functions. *LINC00881* (long intergenic non-protein coding RNA 881, adjusted *p*-value 9.41E−03) was identified as an essential regulator of sarcomere and calcium channel gene expression including *MYH6, CACNA1C*, *RYR2*, and *LINC00881* knockdown was shown to significantly reduce peak calcium amplitude in beating cells^33^. *BANCR* (BRAF-activated non-protein coding RNA, adjusted *p*-value 1.85E−05) was shown to be expressed in fetal heart and pluripotent stem-cell-derived cardiomyocytes, and functional studies also revealed that *BANCR* promoted cardiomyocyte migration in vitro and ventricular enlargement in vivo^34^. Many of the altered lncRNA are transcripts without a defined function; further studies are needed to evaluate their possible role in cardiac development and function. In addition, proper methods for lncRNA sequencing such as library preparation and sequencing depth setting are needed for future projects related to microgravity in order to confirm these initial findings and perform a network-based analysis to gain a deeper insight.

### RNA-seq analysis comparison between short-term and long-term exposure to space microgravity

We previously described the changes in gene expression during a short-term (3 days) exposure of cardiac progenitors to space microgravity^18^ and in this study, we examined the effect of a long-term (3 weeks) exposure to space microgravity. By comparing the RNA-seq data from long-term exposure to space microgravity with those from short-term exposure, we observed a number of common upregulated and downregulated genes, including 11 upregulated genes and 9 downregulated ones (Supplementary Tables 10 and 11). Among the commonly upregulated genes, we found the aforementioned *CCND2*, which has roles in the cell cycle, proliferation, and cardiac differentiation, and FGFR2 (FGF receptor 2) which by binding with protein FGF10 mediates the differentiation of stem cells into cardiomyocytes^35^. Most of the other upregulated genes have roles related to cardiac function, such as *ATP2B4* which is involved in cardiac contraction and conduction, *MYO18B* (Myosin-18B) which binds actin thin filaments and is incorporated in sarcomeres of cardiomyocytes, and *TFRC* (Transferrin receptor) which promotes iron uptake through the transferrin cycle essential for cardiac function^36^. The common downregulated genes show that microgravity affected the extracellular matrix regulation, including the aforementioned downregulated gene *LIMA1* which is involved in focal adhesion while *EFEMP1* (EGF containing fibulin extracellular matrix protein 1) encodes Fibulin-3, an extracellular matrix glycoprotein. A deficiency of Fibulin-3 was recently shown to protect cardiac spheroids against cardiac fibrosis and improving their vascular network^37^. Other downregulated genes are involved in cell senescence and apoptosis, like *TKT* (Transketolase) which promotes cardiomyocyte apoptosis via the cleaved Parp1/Aif pathway^38^ and DNA-damage inducible transcript 3 (*DDIT3*) which is involved in endoplasmic reticulum protein processing and plays a role in cardiomyocyte senescence^39^. These results suggest that even short-term exposure to microgravity improved cardiomyocyte differentiation, cell survivability, and cardiac functions and that some of those changes were not temporary but maintained in the long-term ISS cultures.

## Discussion

Following transcriptomics analyses of live cell samples, we found that 3D cardiac spheres that were under long-term space microgravity (3 weeks on the ISS) were in a state of increased cell growth and cardiac differentiation compared with the ISS 1G control samples. We also discovered that long-term exposure to space microgravity resulted in gene expression profiles of enhanced cardiac development and function. Additionally, the downregulation of genes associated with extracellular matrix was observed in the ISS µG samples compared with the ISS 1G samples. Our conclusions are based on the results of DEGs identified by RNA-seq together with both the GO term analysis and the KEGG pathway mapping.

These effects of long-term exposure to space microgravity on hiPSC-CMs were also consistent with the previous findings on 3-day (short-term) exposure to space microgravity^18^. In our previous study, we also examined by quantitative RT-PCR analysis of the expression of genes related to cell cycle and cell proliferation (such as *CCND1* and *CCND2*), cardiac structure (such as *MYL2 and TNNI3*), and Ca^2+^ handling (such as *ATP2B4*) in both short-term and long-term ISS cultures. We confirmed that the changes in the expression of these genes observed in short-term exposure to microgravity were also present in long-term exposure samples^18^, which was consistent with gene expression changes detected by RNA-seq in this study.

Beneficial effects of microgravity such as increased proliferation have also been observed in other cell types. Compared with ground-based control, neonatal cardiovascular progenitors cultured aboard the ISS for 30 days had enhanced cell proliferation^40^. Under simulated microgravity, bone marrow-derived human mesenchymal stem cells^41^ and adipose-derived stem cells^42^ had increased cell proliferation. In addition, the culture of human mesenchymal stem cells on the ISS for 7 and 14 days increased the immunosuppressive capacity of the cells, which could facilitate the use of these cells in therapy^43^. Together, our study adds to the currently limited body of research on human cardiac cells in space and on changes in the proliferation and differentiation of cells in microgravity.

An innovative aspect of our project is that cells were cultured at 37 °C using the MVP system on the ISS. The MVP is configured to load multiple cultures under both ISS μG and ISS 1G conditions; the 1G condition on the ISS was achieved by centrifugation of one carousel within the same MVP system. Consequently, it allowed for better characterization of the impact of space microgravity alone on gene expression of stem-cell-derived cardiomyocytes. Unlike a ground control, the 1G condition obtained through the MVP module allowed us to focus only on the effect of space microgravity without any background noise related to the space environment, such as space radiation, that could potentially alter or mask the effect of microgravity on gene expression. Therefore, the effects of space microgravity on the growth and development of cardiac progenitors we observed were independent of other potential external factors that might also affect cultures on the ISS.

Our RNA-seq results indicate enhanced cardiac differentiation. For instance, we detected upregulation of genes known to be involved with cardiac development, contraction, and conduction such as *MYL2, TNNI3, SCN5A, ATP2B4*, and *RYR2*. Consistently, the KEGG pathway map of “adrenergic signaling in cardiomyocytes” shows a similar pattern of gene expression necessary for proper cardiomyocyte electrophysiology and contraction. These results are consistent with our observation that space microgravity improved intracellular Ca^2+^ handling in hiPSC-CMs^18^. In addition, increased expression of Ca^2+^ handling and signaling genes has been observed in human cardiovascular progenitors following spaceflight^13,44^. Increased expression of genes associated with mitochondrial metabolism and cardiac structural proteins has also been observed in 2D cultures of late-stage hiPSC-CMs following spaceflight^12^. Together, these results indicate that space microgravity can potentially affect the function of cardiac cells, although additional functional studies are needed for the quantification of the effects of microgravity.

Our results also suggest that exposure to space microgravity could lead to enhanced cell proliferation. For example, we detected upregulation of genes involved with the regulation of cell division such as *KIF14*, *IGF2*, *KIF18B*, *SUSD2*, *RBL1*, and *AURKB*. While not included in any specific GO terms associated with proliferation, upregulation of cell cycle regulators *CCND1*, *CCND2*, *IGF2*, and *TBX3* were also detected. These genes play a role in cell cycle, cell proliferation, and heart regeneration^45–48^. For example, in a swine model of myocardial infarction, *CCND2* overexpression in hiPSC-CMs enhances myocardial repair^49^. In addition, we observed that the expression of *CCND1*, *CCND2*, *IGF2*, and *TBX3* was highly upregulated in cardiac progenitor cells subjected to both a short-term exposure (3 days) and a long-term (3 weeks) to space microgravity as detected by quantitative RT-PCR^18^, further confirming their role in cardiac differentiation and proliferation. *CCND2* together with other cell cycle regulator genes identified in this study could be interesting targets for future investigation since factors that stimulate the proliferation of cardiomyocytes or cardiac progenitors could help to enhance the generation of cardiomyocytes from hiPSCs and facilitate their application in basic and translational studies. Therefore, the ISS experiments are valuable for understanding the mechanisms to generate functional cardiomyocytes under 1G conditions in a more efficient way for different applications. Future studies are needed to focus more on mechanistic aspects of the cardiomyogenic effects of microgravity, since elucidation of the mechanisms may contribute to generating functional cardiomyocytes under 1G conditions.

In addition to GO analysis, KEGG pathway maps were useful for visualizing underlying processes that may contribute to the overall transcriptomic profile. Adrenergic signaling pathway mapping, for instance, was helpful in confirming one of the underlying mechanisms by which the ISS µG hiPSC-CMs were able to show enhanced cardiac function. Likewise, downregulated genes in the KEGG pathway of ECM-receptor interaction were also consistent with what was observed from GO analysis in terms of ECM-related terms and focal adhesion. Whether the downregulation of genes in ECM-term and focal adhesion is relevant to cell proliferation and cardiac development in the context of in vitro 3D hiPSC-CM system remains an interesting question, since ECM downregulation has been previously reported to promote cardiac dedifferentiation and proliferation by regulating mechanical stiffness of its microenvironment^50^.

In this study, we investigated the impact of long-term space microgravity on the transcriptome of hiPSC-CMs by comparing cultures under the ISS µG condition with the ISS 1 G condition. The 3-week cultures on the ISS revealed interesting changes in their transcriptome but were not without limitations. First, space experiment conducted on the ISS poses a common challenge of limited samples retrieved for further analysis. Greater numbers of replicates and cells returned would be desirable for future studies to better understand the functional implications of DEGs observed. Nevertheless, the role of outstanding DEGs identified in this study remains an interesting target for future research. Second, the cellular composition of our 3D cultures contained mostly cardiomyocytes^18^ and the role of cardiac fibroblasts in this process is lesser known. One interesting question to address in a future study may be to better understand the interaction occurring between cardiac cells and non-cardiac cells in 3D hiPSC-CMs, and how the cell composition may change in extended culture in space. Third, a more advanced cell culture module with improved imaging and other characterization tools would help retrieve more visual data during cell culture and between medium changes. For such purposes, future improvements in flight hardware with automated and more advanced imaging systems and other characterization tools would be desirable.

In conclusion, our transcriptomics analyses provide insight into the developmental process of cryopreserved 3D cardiac progenitors under extended exposure to space microgravity. Our findings suggest that the combination of microgravity and the 3D culture leads to increased differentiation and proliferation of cardiac progenitors, which could lead to highly desired future applications of hiPSC-CMs in therapy. Furthermore, genes and pathways identified here may be targets for future research on Earth to mimic the effects of space microgravity.

## Methods

### Cell culture and cryopreservation of cardiac progenitor spheres

For the spaceflight experiment, we prepared cryopreserved cardiac progenitor spheres from IMR90 hiPSC lines (WiCell Research Institute) that were cultured in a feeder-free condition on Matrigel-coated plates and daily fed with mTeSR1 medium. For the induction of IMR90 cardiomyocyte differentiation, hiPSCs were treated with growth factors^51,52^. Briefly, when compact colonies reached >95% confluence, cells were treated with 100 ng/mL activin A from differentiation day 0 till day 1 and then 10 ng/mL bone morphogenic protein-4 (BMP4) from day 1 till day 4 in RPMI/B27 insulin-free medium (RPMI 1640 with 2% B27 minus insulin). Cells were maintained in RPMI/B27 medium (RPMI 1640 with 2% B27 supplement with insulin) until differentiation day 6 when cardiac progenitor spheres were generated using microscale tissue engineering using Aggrewell 400 plates (STEMCELL Technologies)^53^. Briefly, cells were dissociated using 0.25% trypsin-EDTA (Thermo Fisher Scientific), resuspended for cell counting, then seeded into the Aggrewell 400 plates at a concentration of 1.8 × 10^6^ cells/well (1500 cells/microwell). Cells were cultured in RPMI/B27 medium and 10 μM ROCK inhibitor Y-27632 to facilitate cell survival following dissociation^54^. After 24 h, cardiac spheres were cryopreserved.

For cryopreservation, cardiac progenitor spheres were resuspended in 0.5 mL cryopreservation medium (90% fetal bovine serum and 10% dimethyl sulfoxide with 10 μM ROCK inhibitor) and transferred into cryosyringes at 0.5 mL/cryosyringe. Cardiac spheres were initially pre-cooled at 4 °C for up to 25 min to maximize cryopreservation efficiency and then stored at −80 °C in a cooling box.

### ISS cell culture operation

For thawing cells on the ISS, cryosyringes containing cardiac progenitor spheres were placed in a thermoblock at 37 °C for 5 min. The cells were then injected into cell culture chambers of the MVP modules containing a CO_2_-independent medium with 10 μM ROCK inhibitor^55^. The MVP modules were re-installed into the MVP facility, which started with a medium flush cycle to replace the medium with a new culture medium (20 mL per chamber; ~2× chamber volume), to flush out the DMSO in the cryopreserved cell solution (0.5 mL/cryosyringe). The cells were cultured at 37 °C in the MVP system for 22 days on the ISS with medium exchange every other day.

At the end of the mission, live cultures were returned to the ground via warm storage after having been cultured aboard the ISS. Upon arrival at Emory University, cardiac spheres were transferred immediately into an incubator and let recover overnight. The following day cardiac spheres were transferred from the collection bags into low adhesion dishes. Medium was changed from CO_2_-independent medium to standard cardiomyocyte culture medium (RPMI/B27 medium), and cardiac spheres were then maintained overnight, followed by RNA isolation.

### RNA-seq analyses

RNA was isolated using RNeasy Mini Kit (Qiagen) as per the manufacturer’s instructions. RNA concentration was measured using a NanoDrop ND-1000 spectrophotometer (Thermo Fisher Scientific). RNA sequencing, quality control, and transcriptome mapping were done by the Yerkes National Primate Research Center of Emory University. Total RNA quality was tested using an Agilent 4200 TapeStation and RNA 6000 Nano and Pico Chip (Agilent Technologies). RNA samples of triplicate ISS µG and ISS 1G cultures collected after long-term exposure to space microgravity were subjected to library preparation and sequencing.

Two nanograms of total RNA were used as input for cDNA synthesis, using the Clontech SMART-Seq v4 Ultra Low Input RNA kit (Takara Bio) according to the manufacturer’s instructions. Amplified cDNA was fragmented and appended with dual-indexed bar codes using the NexteraXT DNA Library Preparation kit (Illumina). Libraries were validated by capillary electrophoresis on an Agilent 4200 TapeStation, pooled at equimolar concentrations, and sequenced on an Illumina NovaSeq 6000 at 100SR, yielding an average of 30 million reads per sample. Alignment was performed using STAR version 2.7.3a and transcripts were annotated using GRCh38. Transcript abundance estimates were calculated internally to the STAR aligner using the algorithm of htseq-count^56^.

All downstream analyses were performed in R 4.1.2. Read count normalization and differential expression analyses were performed using DESeq2 R package 1.34.0^57^. Gene ontology (GO) and Kyoto Encyclopedia of Genes and Genomes (KEGG) pathway enrichment analyses of differentially expressed genes were done with a default *p*-value cutoff of 0.05 using clusterProfiler R package 4.2.2 and Chord diagrams were generated using GOplot 1.0.2^58,59^.

### Supplementary information

Supplemental material

## Data Availability

RNA-seq data reported in this paper is available at GEO: GEO accession number GSE228063.
