# Supplementary material for: Space microgravity increases expression of genes associated with proliferation and differentiation in human cardiac spheres
Source: NPJ Microgravity. 2023 Dec 9;9:88. doi: 10.1038/s41526-023-00336-6 (PMC10710480; doi:10.1038/s41526-023-00336-6)
Supplement: Supplementary file 1 — Supplemental material [file 41526_2023_336_MOESM1_ESM.pdf]

Supplementary Information

Space microgravity increases expression of genes associated with proliferation and differentiation in human cardiac spheres

Hyun Hwang[1, *], Antonio Rampoldi[1, *], Parvin Forghani[1], Dong Li[1], Jordan Fite[2], Gene Boland[2], Kevin Maher[1], and Chunhui Xu[1,3]

[1]Department of Pediatrics, Emory University School of Medicine and Children's Healthcare of Atlanta, Atlanta, GA, USA

[2] Techshot, Inc., Greenville, IN, USA

[3]Wallace H. Coulter Department of Biomedical Engineering, Georgia Institute of Technology and Emory University, Atlanta, GA, USA

* These authors made equal contributions.

Correspondence: Chunhui Xu, PhD, Professor, Department of Pediatrics, Emory University School of Medicine. Email: chunhui.xu@emory.edu.

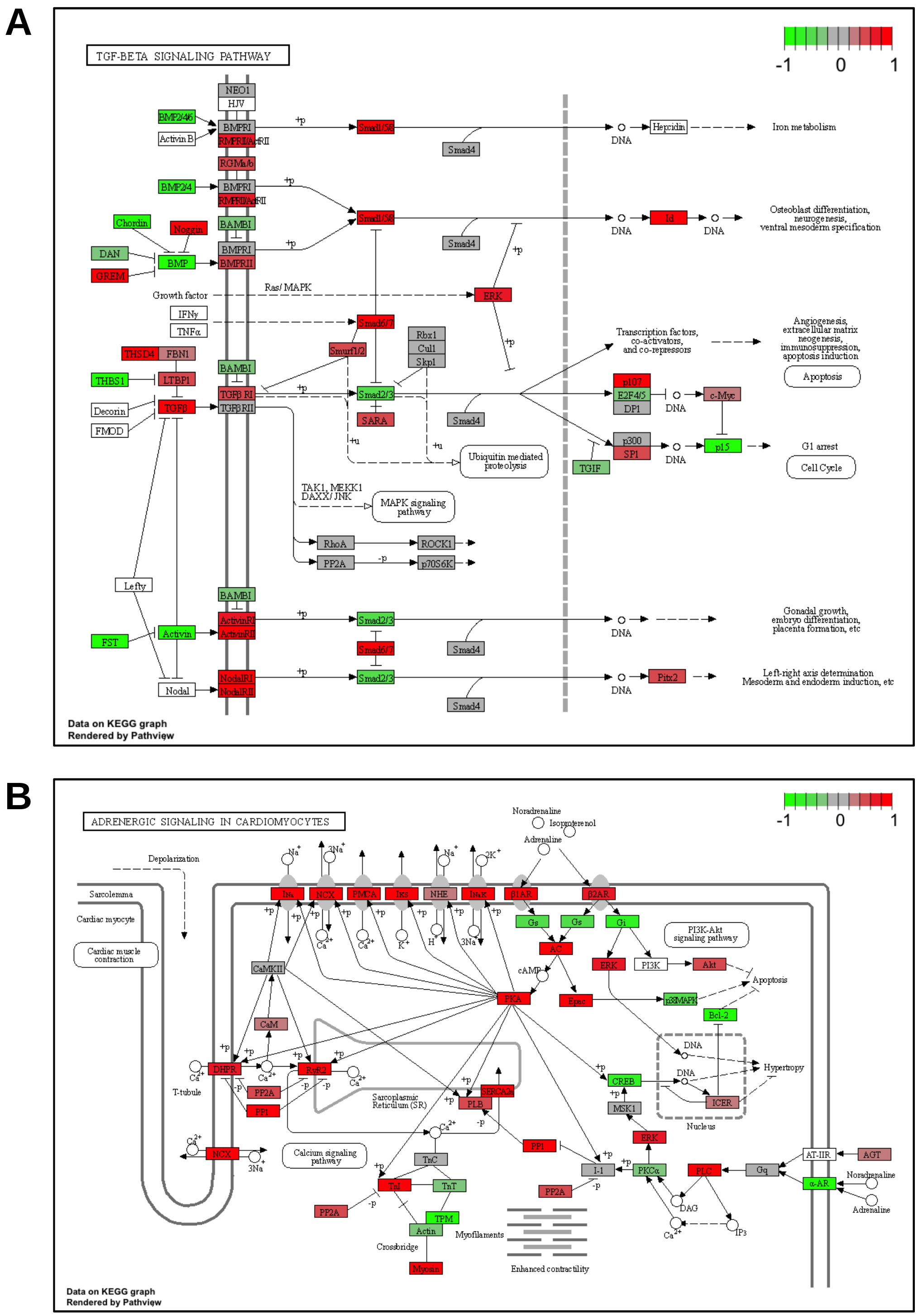

Supplementary Figure 1. KEGG mapping of TGF-beta signaling and adrenergic signaling pathways. Pathway maps were derived from KEGG analysis using our RNA-seq data set. Each gene is color-coded by the KEGG analysis, indicating the level of expression upregulated or downregulated by space microgravity. (A) TGF-beta signaling pathway in IMR90 cardiac spheres after 3 weeks in the ISS µG condition compared with the ISS 1G condition. (B) Adrenergic signaling pathway. KEGG, Kyoto Encyclopedia of Genes and Genomes. ISS 1G, the 1G condition on the International Space Station; ISS µG, the microgravity condition on the International Space Station.

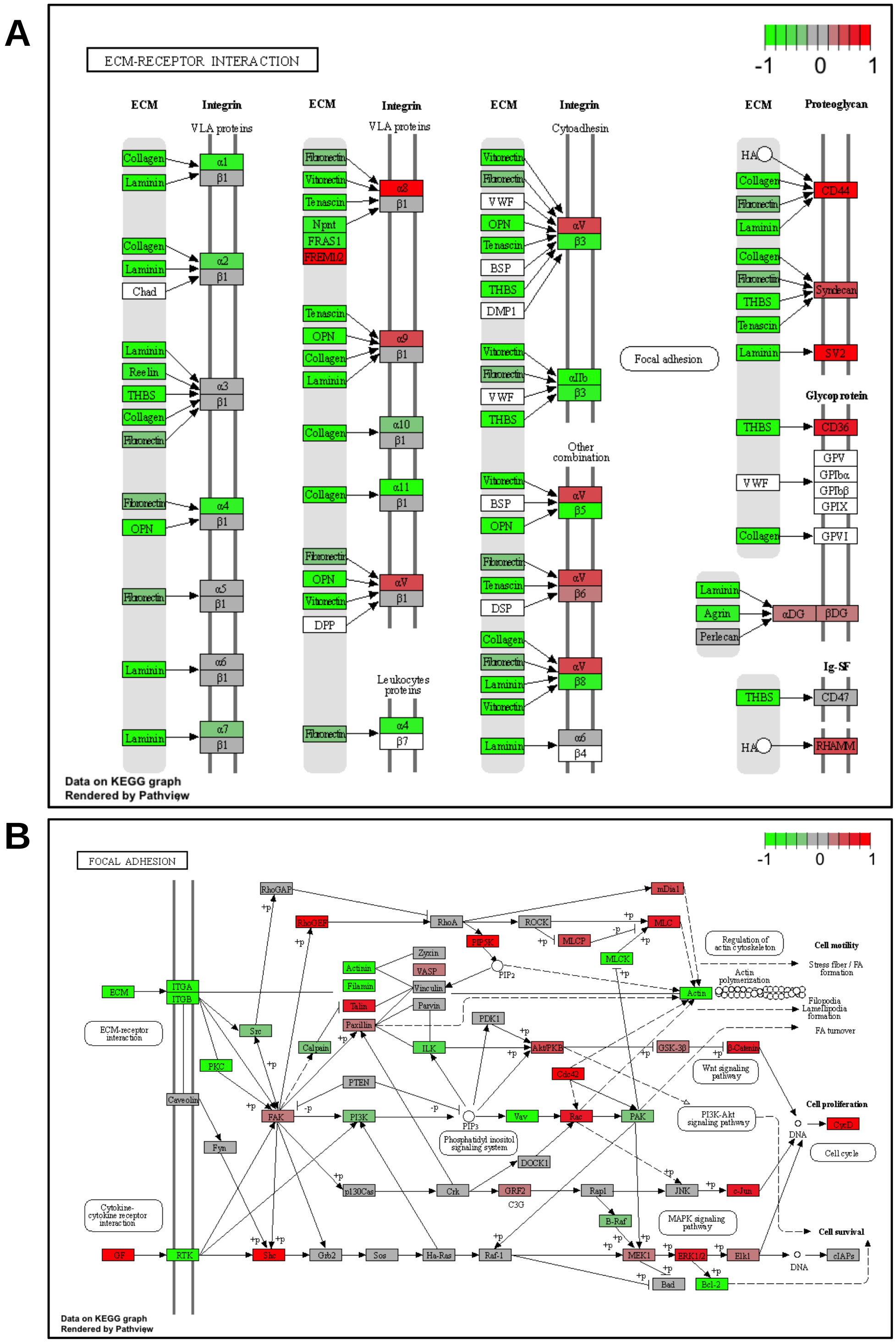

Supplementary Figure 2. KEGG mapping of ECM-receptor interaction and focal adhesion pathways. Pathway maps were derived from KEGG analysis using our RNA-seq data set. Each gene is color-coded by the KEGG analysis, indicating the level of expression upregulated or downregulated by space microgravity. (A) ECM-receptor interaction pathway in IMR90 cardiac spheres after 3 weeks in the ISS µG condition compared with the ISS 1G condition. (B) Focal adhesion pathway. KEGG, Kyoto Encyclopedia of Genes and Genomes. ISS 1G, the 1G condition on the International Space Station; ISS µG, the microgravity condition on the International Space Station.

Supplementary Table 1. 271 upregulated differentially expressed genes (DEGs) in the ISS μG condition compared with the ISS 1G condition.

Gene	Description	log2(Fold change)	adjusted p-value
SLC5A9	solute carrier family 5 member 9 [Source:HGNC Symbol;Acc:HGNC:22146]	6.99	6.70E-03
BDKRB2	bradykinin receptor B2 [Source:HGNC Symbol;Acc:HGNC:1030]	6.87	4.13E-03
GADL1	glutamate decarboxylase like 1 [Source:HGNC Symbol;Acc:HGNC:27949]	6.20	3.81E-02
THBD	thrombomodulin [Source:HGNC Symbol;Acc:HGNC:11784]	5.76	1.31E-04
AQP5	aquaporin 5 [Source:HGNC Symbol;Acc:HGNC:638]	5.00	1.08E-02
UPK1A	uroplakin 1A [Source:HGNC Symbol;Acc:HGNC:12577]	4.74	4.93E-02
COL11A1	collagen type XI alpha 1 chain [Source:HGNC Symbol;Acc:HGNC:2186]	4.67	7.71E-04
CCBE1	collagen and calcium binding EGF domains 1 [Source:HGNC Symbol;Acc:HGNC:29426]	3.75	7.67E-12
SOCS3	suppressor of cytokine signaling 3 [Source:HGNC Symbol;Acc:HGNC:19391]	3.69	1.22E-05
SNCB	synuclein beta [Source:HGNC Symbol;Acc:HGNC:11140]	3.65	2.90E-02
CTNNA2	catenin alpha 2 [Source:HGNC Symbol;Acc:HGNC:2510]	3.53	3.61E-09
ATOH8	atonal bHLH transcription factor 8 [Source:HGNC Symbol;Acc:HGNC:24126]	3.33	8.27E-05
SLC16A3	solute carrier family 16 member 3 [Source:HGNC Symbol;Acc:HGNC:10924]	3.24	4.91E-02
TRPA1	transient receptor potential cation channel subfamily A member 1 [Source:HGNC Symbol;Acc:HGNC:497]	3.08	4.91E-02
SPAG4	sperm associated antigen 4 [Source:HGNC Symbol;Acc:HGNC:11214]	2.98	2.78E-03
PNOC	prepronociceptin [Source:HGNC Symbol;Acc:HGNC:9163]	2.85	5.74E-04
BRIP1	BRCA1 interacting protein C-terminal helicase 1 [Source:HGNC Symbol;Acc:HGNC:20473]	2.78	1.95E-02
SEC14L6	SEC14 like lipid binding 6 [Source:HGNC Symbol;Acc:HGNC:40047]	2.71	4.85E-03
SHROOM4	shroom family member 4 [Source:HGNC Symbol;Acc:HGNC:29215]	2.57	3.60E-06
TAFA2	TAFA chemokine like family member 2 [Source:HGNC Symbol;Acc:HGNC:21589]	2.50	2.32E-03
MYL2	myosin light chain 2 [Source:HGNC Symbol;Acc:HGNC:7583]	2.44	4.39E-03
SLC26A9	solute carrier family 26 member 9 [Source:HGNC Symbol;Acc:HGNC:14469]	2.41	1.50E-07
EGR4	early growth response 4 [Source:HGNC Symbol;Acc:HGNC:3241]	2.34	5.66E-03
ADRA2B	adrenoceptor alpha 2B [Source:HGNC Symbol;Acc:HGNC:282]	2.24	7.31E-03
IGFBP5	insulin like growth factor binding protein 5 [Source:HGNC Symbol;Acc:HGNC:5474]	2.19	6.94E-10
RIN3	Ras and Rab interactor 3 [Source:HGNC Symbol;Acc:HGNC:18751]	2.17	3.87E-02
MT3	metallothionein 3 [Source:HGNC Symbol;Acc:HGNC:7408]	2.13	3.73E-03
KIF14	kinesin family member 14 [Source:HGNC Symbol;Acc:HGNC:19181]	2.11	2.42E-03
IGF2	insulin like growth factor 2 [Source:HGNC Symbol;Acc:HGNC:5466]	2.07	9.89E-03
ANKRD63	ankyrin repeat domain 63 [Source:HGNC Symbol;Acc:HGNC:40027]	2.06	1.77E-02
ADPRHL1	ADP-ribosylhydrolase like 1 [Source:HGNC Symbol;Acc:HGNC:21303]	2.06	4.28E-09
TMEM200B	transmembrane protein 200B [Source:HGNC Symbol;Acc:HGNC:33785]	2.05	1.44E-02
ACTN3	actinin alpha 3 (gene/pseudogene) [Source:HGNC Symbol;Acc:HGNC:165]	2.04	1.87E-07

SORL1	sortilin related receptor 1 [Source:HGNC Symbol;Acc:HGNC:11185]	2.04	3.97E-08
KCNQ3	potassium voltage-gated channel subfamily Q member 3 [Source:HGNC Symbol;Acc:HGNC:6297]	2.03	1.95E-02
LOXL3	lysyl oxidase like 3 [Source:HGNC Symbol;Acc:HGNC:13869]	2.02	3.64E-04
MIR210HG	MIR210 host gene [Source:HGNC Symbol;Acc:HGNC:39524]	2.02	3.38E-04
LINC00880	long intergenic non-protein coding RNA 880 [Source:HGNC Symbol;Acc:HGNC:27948]	2.01	2.45E-03
CCND2	cyclin D2 [Source:HGNC Symbol;Acc:HGNC:1583]	2.01	4.41E-11
ADAMTS17	ADAM metallopeptidase with thrombospondin type 1 motif 17 [Source:HGNC Symbol;Acc:HGNC:17109]	2.00	4.44E-02
LDHAP4	lactate dehydrogenase A pseudogene 4 [Source:HGNC Symbol;Acc:HGNC:6539]	2.00	1.46E-02
EGLN3	egl-9 family hypoxia inducible factor 3 [Source:HGNC Symbol;Acc:HGNC:14661]	1.95	1.66E-05
AC107068.2	novel transcript, antisense to CORIN and NFXL1	1.95	3.31E-03
ID1	inhibitor of DNA binding 1, HLH protein [Source:HGNC Symbol;Acc:HGNC:5360]	1.89	6.10E-03
KIF18B	kinesin family member 18B [Source:HGNC Symbol;Acc:HGNC:27102]	1.89	3.23E-02
SUSD2	sushi domain containing 2 [Source:HGNC Symbol;Acc:HGNC:30667]	1.88	2.86E-08
NR4A1	nuclear receptor subfamily 4 group A member 1 [Source:HGNC Symbol;Acc:HGNC:7980]	1.87	6.94E-10
IL4R	interleukin 4 receptor [Source:HGNC Symbol;Acc:HGNC:6015]	1.86	6.31E-07
KCNN1	potassium calcium-activated channel subfamily N member 1 [Source:HGNC Symbol;Acc:HGNC:6290]	1.82	7.53E-03
FOXM1	forkhead box M1 [Source:HGNC Symbol;Acc:HGNC:3818]	1.80	1.39E-02
NEK2	NIMA related kinase 2 [Source:HGNC Symbol;Acc:HGNC:7745]	1.78	1.93E-02
CDCA2	cell division cycle associated 2 [Source:HGNC Symbol;Acc:HGNC:14623]	1.77	1.34E-02
LINC01470	long intergenic non-protein coding RNA 1470 [Source:HGNC Symbol;Acc:HGNC:51105]	1.74	5.21E-03
AC015522.1	novel transcript	1.72	1.61E-02
VWC2	von Willebrand factor C domain containing 2 [Source:HGNC Symbol;Acc:HGNC:30200]	1.70	1.81E-02
PDCD6IPP2	PDCD6IP pseudogene 2 [Source:HGNC Symbol;Acc:HGNC:49873]	1.69	3.85E-02
ID3	inhibitor of DNA binding 3, HLH protein [Source:HGNC Symbol;Acc:HGNC:5362]	1.68	1.50E-07
OPCML	opioid binding protein/cell adhesion molecule like [Source:HGNC Symbol;Acc:HGNC:8143]	1.68	3.54E-02
AC021678.2	novel transcript	1.67	1.53E-02
TNNI3	troponin I3, cardiac type [Source:HGNC Symbol;Acc:HGNC:11947]	1.64	2.58E-09
GCNT1	glucosaminyl (N-acetyl) transferase 1 [Source:HGNC Symbol;Acc:HGNC:4203]	1.59	4.93E-03
FAIM2	Fas apoptotic inhibitory molecule 2 [Source:HGNC Symbol;Acc:HGNC:17067]	1.59	3.89E-02
GREM2	gremlin 2, DAN family BMP antagonist [Source:HGNC Symbol;Acc:HGNC:17655]	1.59	1.57E-02
PRUNE2	prune homolog 2 with BCH domain [Source:HGNC Symbol;Acc:HGNC:25209]	1.57	1.20E-05
ARHGAP45	Rho GTPase activating protein 45 [Source:HGNC Symbol;Acc:HGNC:17102]	1.56	9.78E-03
CDH13	cadherin 13 [Source:HGNC Symbol;Acc:HGNC:1753]	1.55	1.32E-02

ERCC6L	ERCC excision repair 6 like, spindle assembly checkpoint helicase [Source:HGNC Symbol;Acc:HGNC:20794]	1.55	2.69E-02
STARD13	StAR related lipid transfer domain containing 13 [Source:HGNC Symbol;Acc:HGNC:19164]	1.53	5.24E-04
APOLD1	apolipoprotein L domain containing 1 [Source:HGNC Symbol;Acc:HGNC:25268]	1.53	2.29E-02
ARHGEF37	Rho guanine nucleotide exchange factor 37 [Source:HGNC Symbol;Acc:HGNC:34430]	1.53	1.22E-05
MAB21L3	mab-21 like 3 [Source:HGNC Symbol;Acc:HGNC:26787]	1.51	3.87E-02
KIFC1	kinesin family member C1 [Source:HGNC Symbol;Acc:HGNC:6389]	1.50	1.09E-02
LMNB1	lamin B1 [Source:HGNC Symbol;Acc:HGNC:6637]	1.49	1.55E-02
HHATL	hedgehog acyltransferase like [Source:HGNC Symbol;Acc:HGNC:13242]	1.49	3.60E-06
SSPN	sarcospan [Source:HGNC Symbol;Acc:HGNC:11322]	1.47	1.33E-02
NFIB	nuclear factor I B [Source:HGNC Symbol;Acc:HGNC:7785]	1.42	2.14E-02
TOP2A	DNA topoisomerase II alpha [Source:HGNC Symbol;Acc:HGNC:11989]	1.41	3.24E-03
C2orf88	chromosome 2 open reading frame 88 [Source:HGNC Symbol;Acc:HGNC:28191]	1.40	1.28E-04
KLHL29	kelch like family member 29 [Source:HGNC Symbol;Acc:HGNC:29404]	1.38	3.27E-02
ASB11	ankyrin repeat and SOCS box containing 11 [Source:HGNC Symbol;Acc:HGNC:17186]	1.35	3.70E-03
BCL11A	BAF chromatin remodeling complex subunit BCL11A [Source:HGNC Symbol;Acc:HGNC:13221]	1.34	3.27E-02
ARTN	artemin [Source:HGNC Symbol;Acc:HGNC:727]	1.34	8.95E-03
NMRK2	nicotinamide riboside kinase 2 [Source:HGNC Symbol;Acc:HGNC:17871]	1.34	1.34E-07
H1-0	H1.0 linker histone [Source:HGNC Symbol;Acc:HGNC:4714]	1.34	2.75E-05
H4C3	H4 clustered histone 3 [Source:HGNC Symbol;Acc:HGNC:4787]	1.34	3.92E-11
TRH	thyrotropin releasing hormone [Source:HGNC Symbol;Acc:HGNC:12298]	1.33	2.14E-03
PLEKHA4	pleckstrin homology domain containing A4 [Source:HGNC Symbol;Acc:HGNC:14339]	1.33	2.40E-06
NLGN1	neuroligin 1 [Source:HGNC Symbol;Acc:HGNC:14291]	1.33	2.14E-03
LRRC14B	leucine rich repeat containing 14B [Source:HGNC Symbol;Acc:HGNC:37268]	1.32	7.57E-04
RBL1	RB transcriptional corepressor like 1 [Source:HGNC Symbol;Acc:HGNC:9893]	1.32	1.19E-02
CCDC151	coiled-coil domain containing 151 [Source:HGNC Symbol;Acc:HGNC:28303]	1.32	2.76E-02
SCD5	stearoyl-CoA desaturase 5 [Source:HGNC Symbol;Acc:HGNC:21088]	1.31	3.15E-02
FGF1	fibroblast growth factor 1 [Source:HGNC Symbol;Acc:HGNC:3665]	1.30	5.54E-07
IER2	immediate early response 2 [Source:HGNC Symbol;Acc:HGNC:28871]	1.29	5.05E-03
SMCO1	single-pass membrane protein with coiled-coil domains 1 [Source:HGNC Symbol;Acc:HGNC:27407]	1.28	2.79E-04
AURKB	aurora kinase B [Source:HGNC Symbol;Acc:HGNC:11390]	1.28	2.47E-02
OLFML2A	olfactomedin like 2A [Source:HGNC Symbol;Acc:HGNC:27270]	1.27	1.15E-02
FGF16	fibroblast growth factor 16 [Source:HGNC Symbol;Acc:HGNC:3672]	1.23	3.50E-02
RGS9	regulator of G protein signaling 9 [Source:HGNC Symbol;Acc:HGNC:10004]	1.22	3.36E-04
PRRG3	proline rich and Gla domain 3 [Source:HGNC Symbol;Acc:HGNC:30798]	1.21	1.41E-02
ATP2B4	ATPase plasma membrane Ca2+ transporting 4 [Source:HGNC Symbol;Acc:HGNC:817]	1.20	3.31E-03

CCNB2	cyclin B2 [Source:HGNC Symbol;Acc:HGNC:1580]	1.19	4.58E-02
ZFP36	ZFP36 ring finger protein [Source:HGNC Symbol;Acc:HGNC:12862]	1.19	6.71E-03
PGK1	phosphoglycerate kinase 1 [Source:HGNC Symbol;Acc:HGNC:8896]	1.18	4.93E-03
TSPAN9	tetraspanin 9 [Source:HGNC Symbol;Acc:HGNC:21640]	1.16	4.55E-03
THSD4	thrombospondin type 1 domain containing 4 [Source:HGNC Symbol;Acc:HGNC:25835]	1.16	1.48E-03
SMAD6	SMAD family member 6 [Source:HGNC Symbol;Acc:HGNC:6772]	1.16	9.62E-05
LRRC52-AS1	LRRC52 antisense RNA 1 [Source:HGNC Symbol;Acc:HGNC:54044]	1.16	1.69E-02
ADAMTSL1	ADAMTS like 1 [Source:HGNC Symbol;Acc:HGNC:14632]	1.15	1.96E-02
A2M	alpha-2-macroglobulin [Source:HGNC Symbol;Acc:HGNC:7]	1.15	2.47E-05
PPP1R3A	protein phosphatase 1 regulatory subunit 3A [Source:HGNC Symbol;Acc:HGNC:9291]	1.15	6.23E-05
SOX11	SRY-box transcription factor 11 [Source:HGNC Symbol;Acc:HGNC:11191]	1.15	2.72E-02
PPFIA4	PTPRF interacting protein alpha 4 [Source:HGNC Symbol;Acc:HGNC:9248]	1.15	1.53E-03
EDNRB	endothelin receptor type B [Source:HGNC Symbol;Acc:HGNC:3180]	1.15	6.90E-03
VSNL1	visinin like 1 [Source:HGNC Symbol;Acc:HGNC:12722]	1.14	3.87E-02
B3GNT9	UDP-GlcNAc:betaGal beta-1,3-N-acetylglucosaminyltransferase 9 [Source:HGNC Symbol;Acc:HGNC:28714]	1.13	8.80E-03
TRPC3	transient receptor potential cation channel subfamily C member 3 [Source:HGNC Symbol;Acc:HGNC:12335]	1.12	8.80E-03
P4HA1	prolyl 4-hydroxylase subunit alpha 1 [Source:HGNC Symbol;Acc:HGNC:8546]	1.12	1.31E-02
FXYD6	FXYD domain containing ion transport regulator 6 [Source:HGNC Symbol;Acc:HGNC:4030]	1.11	6.79E-03
CENPF	centromere protein F [Source:HGNC Symbol;Acc:HGNC:1857]	1.11	3.81E-02
SDK2	sidekick cell adhesion molecule 2 [Source:HGNC Symbol;Acc:HGNC:19308]	1.11	1.69E-03
SMAD7	SMAD family member 7 [Source:HGNC Symbol;Acc:HGNC:6773]	1.10	2.81E-04
TSPAN15	tetraspanin 15 [Source:HGNC Symbol;Acc:HGNC:23298]	1.09	4.72E-02
HS3ST3B1	heparan sulfate-glucosamine 3-sulfotransferase 3B1 [Source:HGNC Symbol;Acc:HGNC:5198]	1.09	2.31E-02
BIRC5	baculoviral IAP repeat containing 5 [Source:HGNC Symbol;Acc:HGNC:593]	1.09	3.13E-02
WDHD1	WD repeat and HMG-box DNA binding protein 1 [Source:HGNC Symbol;Acc:HGNC:23170]	1.08	1.11E-02
NFATC2	nuclear factor of activated T cells 2 [Source:HGNC Symbol;Acc:HGNC:7776]	1.08	2.67E-02
PDK1	pyruvate dehydrogenase kinase 1 [Source:HGNC Symbol;Acc:HGNC:8809]	1.08	1.08E-02
ATP1A1	ATPase Na+/K+ transporting subunit alpha 1 [Source:HGNC Symbol;Acc:HGNC:799]	1.08	3.00E-03
NUSAP1	nucleolar and spindle associated protein 1 [Source:HGNC Symbol;Acc:HGNC:18538]	1.08	1.47E-02
EPN3	epsin 3 [Source:HGNC Symbol;Acc:HGNC:18235]	1.06	2.04E-02
PRICKLE2	prickle planar cell polarity protein 2 [Source:HGNC Symbol;Acc:HGNC:20340]	1.06	1.75E-04
PHF19	PHD finger protein 19 [Source:HGNC Symbol;Acc:HGNC:24566]	1.06	2.90E-02
INTS2	integrator complex subunit 2 [Source:HGNC Symbol;Acc:HGNC:29241]	1.05	3.21E-02
C14orf132	chromosome 14 open reading frame 132 [Source:HGNC Symbol;Acc:HGNC:20346]	1.05	1.33E-02

ST8SIA2	ST8 alpha-N-acetyl-neuraminide alpha-2,8-sialyltransferase 2 [Source:HGNC Symbol;Acc:HGNC:10870]	1.05	4.69E-02
FUT11	fucosyltransferase 11 [Source:HGNC Symbol;Acc:HGNC:19233]	1.04	3.36E-02
ACVR2B	activin A receptor type 2B [Source:HGNC Symbol;Acc:HGNC:174]	1.04	3.28E-02
PLXNA4	plexin A4 [Source:HGNC Symbol;Acc:HGNC:9102]	1.04	1.29E-02
CKM	creatine kinase, M-type [Source:HGNC Symbol;Acc:HGNC:1994]	1.04	2.86E-08
FAM162A	family with sequence similarity 162 member A [Source:HGNC Symbol;Acc:HGNC:17865]	1.04	2.42E-03
KCNQ1	potassium voltage-gated channel subfamily Q member 1 [Source:HGNC Symbol;Acc:HGNC:6294]	1.04	1.67E-02
MB	myoglobin [Source:HGNC Symbol;Acc:HGNC:6915]	1.03	1.55E-02
TBX3	T-box transcription factor 3 [Source:HGNC Symbol;Acc:HGNC:11602]	1.02	5.48E-03
PLCB2	phospholipase C beta 2 [Source:HGNC Symbol;Acc:HGNC:9055]	1.02	4.06E-02
PHACTR1	phosphatase and actin regulator 1 [Source:HGNC Symbol;Acc:HGNC:20990]	1.01	3.73E-03
NUCKS1	nuclear casein kinase and cyclin dependent kinase substrate 1 [Source:HGNC Symbol;Acc:HGNC:29923]	1.01	5.58E-04
STING1	stimulator of interferon response cGAMP interactor 1 [Source:HGNC Symbol;Acc:HGNC:27962]	1.01	4.14E-04
TENM3	teneurin transmembrane protein 3 [Source:HGNC Symbol;Acc:HGNC:29944]	1.00	3.38E-02
DOCK11	dedicator of cytokinesis 11 [Source:HGNC Symbol;Acc:HGNC:23483]	1.00	2.65E-02
CD44	CD44 molecule (Indian blood group) [Source:HGNC Symbol;Acc:HGNC:1681]	1.00	1.05E-02
TIPARP	TCDD inducible poly(ADP-ribose) polymerase [Source:HGNC Symbol;Acc:HGNC:23696]	1.00	5.73E-04
WDR54	WD repeat domain 54 [Source:HGNC Symbol;Acc:HGNC:25770]	0.99	1.14E-02
PLXNA2	plexin A2 [Source:HGNC Symbol;Acc:HGNC:9100]	0.99	7.53E-03
DUSP5	dual specificity phosphatase 5 [Source:HGNC Symbol;Acc:HGNC:3071]	0.99	1.09E-02
ADAMTS9	ADAM metallopeptidase with thrombospondin type 1 motif 9 [Source:HGNC Symbol;Acc:HGNC:13202]	0.98	7.36E-03
LRP5	LDL receptor related protein 5 [Source:HGNC Symbol;Acc:HGNC:6697]	0.98	7.57E-04
CCND1	cyclin D1 [Source:HGNC Symbol;Acc:HGNC:1582]	0.98	1.85E-06
NCALD	neurocalcin delta [Source:HGNC Symbol;Acc:HGNC:7655]	0.97	3.38E-02
LBH	LBH regulator of WNT signaling pathway [Source:HGNC Symbol;Acc:HGNC:29532]	0.97	7.71E-03
MYO18B	myosin XVIIIB [Source:HGNC Symbol;Acc:HGNC:18150]	0.96	2.31E-03
VPS13A	vacuolar protein sorting 13 homolog A [Source:HGNC Symbol;Acc:HGNC:1908]	0.96	1.26E-02
PRKACA	protein kinase cAMP-activated catalytic subunit alpha [Source:HGNC Symbol;Acc:HGNC:9380]	0.95	4.73E-02
GNAO1	G protein subunit alpha o1 [Source:HGNC Symbol;Acc:HGNC:4389]	0.95	4.62E-02
GEM	GTP binding protein overexpressed in skeletal muscle [Source:HGNC Symbol;Acc:HGNC:4234]	0.94	3.41E-02
HNRNPA0	heterogeneous nuclear ribonucleoprotein A0 [Source:HGNC Symbol;Acc:HGNC:5030]	0.94	4.13E-02
JPT2	Jupiter microtubule associated homolog 2 [Source:HGNC Symbol;Acc:HGNC:14137]	0.94	4.18E-02
RRM2	ribonucleotide reductase regulatory subunit M2 [Source:HGNC Symbol;Acc:HGNC:10452]	0.94	3.70E-02

IRF6	interferon regulatory factor 6 [Source:HGNC Symbol;Acc:HGNC:6121]	0.93	4.68E-02
PEX26	peroxisomal biogenesis factor 26 [Source:HGNC Symbol;Acc:HGNC:22965]	0.93	1.72E-02
SYNDIG1	synapse differentiation inducing 1 [Source:HGNC Symbol;Acc:HGNC:15885]	0.93	2.19E-02
PDGFD	platelet derived growth factor D [Source:HGNC Symbol;Acc:HGNC:30620]	0.93	1.28E-02
ETV3	ETS variant transcription factor 3 [Source:HGNC Symbol;Acc:HGNC:3492]	0.92	2.95E-02
TAB3	TGF-beta activated kinase 1 (MAP3K7) binding protein 3 [Source:HGNC Symbol;Acc:HGNC:30681]	0.92	4.16E-02
SRSF6	serine and arginine rich splicing factor 6 [Source:HGNC Symbol;Acc:HGNC:10788]	0.92	3.06E-03
FKBP5	FKBP prolyl isomerase 5 [Source:HGNC Symbol;Acc:HGNC:3721]	0.91	1.49E-02
CDC42	cell division cycle 42 [Source:HGNC Symbol;Acc:HGNC:1736]	0.90	9.44E-04
CBX7	chromobox 7 [Source:HGNC Symbol;Acc:HGNC:1557]	0.90	2.01E-02
THBS4	thrombospondin 4 [Source:HGNC Symbol;Acc:HGNC:11788]	0.90	5.96E-03
KIF1C	kinesin family member 1C [Source:HGNC Symbol;Acc:HGNC:6317]	0.89	1.13E-03
FAM219A	family with sequence similarity 219 member A [Source:HGNC Symbol;Acc:HGNC:19920]	0.89	3.28E-02
RYR2	ryanodine receptor 2 [Source:HGNC Symbol;Acc:HGNC:10484]	0.88	3.29E-03
MSX2	msh homeobox 2 [Source:HGNC Symbol;Acc:HGNC:7392]	0.88	2.46E-03
TFRC	transferrin receptor [Source:HGNC Symbol;Acc:HGNC:11763]	0.88	2.65E-03
SC5D	sterol-C5-desaturase [Source:HGNC Symbol;Acc:HGNC:10547]	0.88	3.22E-03
DMXL2	Dmx like 2 [Source:HGNC Symbol;Acc:HGNC:2938]	0.87	1.79E-03
ZNF385B	zinc finger protein 385B [Source:HGNC Symbol;Acc:HGNC:26332]	0.87	5.17E-03
CREB3L2	cAMP responsive element binding protein 3 like 2 [Source:HGNC Symbol;Acc:HGNC:23720]	0.87	9.86E-03
USP9X	ubiquitin specific peptidase 9 X-linked [Source:HGNC Symbol;Acc:HGNC:12632]	0.86	1.79E-03
SSH2	slingshot protein phosphatase 2 [Source:HGNC Symbol;Acc:HGNC:30580]	0.86	1.25E-02
YEATS2	YEATS domain containing 2 [Source:HGNC Symbol;Acc:HGNC:25489]	0.86	3.29E-03
ABCA2	ATP binding cassette subfamily A member 2 [Source:HGNC Symbol;Acc:HGNC:32]	0.86	5.17E-03
CCDC141	coiled-coil domain containing 141 [Source:HGNC Symbol;Acc:HGNC:26821]	0.86	1.01E-03
TCAP	titin-cap [Source:HGNC Symbol;Acc:HGNC:11610]	0.85	1.11E-03
TXNIP	thioredoxin interacting protein [Source:HGNC Symbol;Acc:HGNC:16952]	0.85	1.55E-02
PLCXD3	phosphatidylinositol specific phospholipase C X domain containing 3 [Source:HGNC Symbol;Acc:HGNC:31822]	0.85	1.94E-02
KDM3A	lysine demethylase 3A [Source:HGNC Symbol;Acc:HGNC:20815]	0.85	7.95E-03
BANCR	BRAF-activated non-protein coding RNA [Source:HGNC Symbol;Acc:HGNC:43877]	0.85	1.85E-05
TMEM65	transmembrane protein 65 [Source:HGNC Symbol;Acc:HGNC:25203]	0.84	3.86E-02
LCLAT1	lysocardiolipin acyltransferase 1 [Source:HGNC Symbol;Acc:HGNC:26756]	0.84	2.37E-02
NEFL	neurofilament light [Source:HGNC Symbol;Acc:HGNC:7739]	0.84	1.69E-02
MCC	MCC regulator of WNT signaling pathway [Source:HGNC Symbol;Acc:HGNC:6935]	0.84	1.85E-05
ADD2	adducin 2 [Source:HGNC Symbol;Acc:HGNC:244]	0.83	2.36E-02

LINC00881	long intergenic non-protein coding RNA 881 [Source:HGNC Symbol;Acc:HGNC:48567]	0.83	9.41E-03
CPNE5	copine 5 [Source:HGNC Symbol;Acc:HGNC:2318]	0.83	4.74E-03
LMOD2	leiomodin 2 [Source:HGNC Symbol;Acc:HGNC:6648]	0.82	4.36E-03
CBX6	chromobox 6 [Source:HGNC Symbol;Acc:HGNC:1556]	0.81	2.63E-02
GNG7	G protein subunit gamma 7 [Source:HGNC Symbol;Acc:HGNC:4410]	0.81	1.71E-02
FAM122B	family with sequence similarity 122B [Source:HGNC Symbol;Acc:HGNC:30490]	0.80	1.23E-02
CACNB1	calcium voltage-gated channel auxiliary subunit beta 1 [Source:HGNC Symbol;Acc:HGNC:1401]	0.79	1.28E-02
ELOVL6	ELOVL fatty acid elongase 6 [Source:HGNC Symbol;Acc:HGNC:15829]	0.79	4.79E-02
ANK2	ankyrin 2 [Source:HGNC Symbol;Acc:HGNC:493]	0.79	4.68E-02
KDM2B	lysine demethylase 2B [Source:HGNC Symbol;Acc:HGNC:13610]	0.78	1.84E-02
DCAF17	DDB1 and CUL4 associated factor 17 [Source:HGNC Symbol;Acc:HGNC:25784]	0.78	4.62E-02
PKM	pyruvate kinase M1/2 [Source:HGNC Symbol;Acc:HGNC:9021]	0.78	9.55E-03
SLC35F1	solute carrier family 35 member F1 [Source:HGNC Symbol;Acc:HGNC:21483]	0.78	3.23E-02
LRRC20	leucine rich repeat containing 20 [Source:HGNC Symbol;Acc:HGNC:23421]	0.78	1.55E-02
PFKL	phosphofructokinase, liver type [Source:HGNC Symbol;Acc:HGNC:8876]	0.77	3.87E-02
TMTC3	transmembrane O-mannosyltransferase targeting cadherins 3 [Source:HGNC Symbol;Acc:HGNC:26899]	0.77	2.25E-03
DGAT2	diacylglycerol O-acyltransferase 2 [Source:HGNC Symbol;Acc:HGNC:16940]	0.77	2.69E-02
MYOM1	myomesin 1 [Source:HGNC Symbol;Acc:HGNC:7613]	0.76	3.27E-02
HEATR1	HEAT repeat containing 1 [Source:HGNC Symbol;Acc:HGNC:25517]	0.76	2.49E-02
DEPP1	DEPP1 autophagy regulator [Source:HGNC Symbol;Acc:HGNC:23355]	0.76	7.53E-03
PDE4DIP	phosphodiesterase 4D interacting protein [Source:HGNC Symbol;Acc:HGNC:15580]	0.76	6.71E-03
ZKSCAN2	zinc finger with KRAB and SCAN domains 2 [Source:HGNC Symbol;Acc:HGNC:25677]	0.74	3.15E-02
C4orf3	chromosome 4 open reading frame 3 [Source:HGNC Symbol;Acc:HGNC:19225]	0.74	3.81E-02
MIDEAS	mitotic deacetylase associated SANT domain protein [Source:HGNC Symbol;Acc:HGNC:19853]	0.74	3.42E-02
MLH3	mutL homolog 3 [Source:HGNC Symbol;Acc:HGNC:7128]	0.73	4.05E-02
C20orf194	chromosome 20 open reading frame 194 [Source:HGNC Symbol;Acc:HGNC:17721]	0.73	1.95E-02
IGF2R	insulin like growth factor 2 receptor [Source:HGNC Symbol;Acc:HGNC:5467]	0.72	1.08E-02
MAPK8IP3	mitogen-activated protein kinase 8 interacting protein 3 [Source:HGNC Symbol;Acc:HGNC:6884]	0.72	2.00E-02
SMCHD1	structural maintenance of chromosomes flexible hinge domain containing 1 [Source:HGNC Symbol;Acc:HGNC:29090]	0.72	2.86E-02
NFYB	nuclear transcription factor Y subunit beta [Source:HGNC Symbol;Acc:HGNC:7805]	0.71	4.58E-02
PRKAA2	protein kinase AMP-activated catalytic subunit alpha 2 [Source:HGNC Symbol;Acc:HGNC:9377]	0.70	1.33E-02
EXOC6B	exocyst complex component 6B [Source:HGNC Symbol;Acc:HGNC:17085]	0.70	3.21E-02
AKAP6	A-kinase anchoring protein 6 [Source:HGNC Symbol;Acc:HGNC:376]	0.69	4.63E-02

PHLDA1	pleckstrin homology like domain family A member 1 [Source:HGNC Symbol;Acc:HGNC:8933]	0.69	4.15E-02
PRSS35	serine protease 35 [Source:HGNC Symbol;Acc:HGNC:21387]	0.68	5.69E-03
CKMT2	creatine kinase, mitochondrial 2 [Source:HGNC Symbol;Acc:HGNC:1996]	0.67	1.47E-02
INSR	insulin receptor [Source:HGNC Symbol;Acc:HGNC:6091]	0.66	1.15E-02
SCN5A	sodium voltage-gated channel alpha subunit 5 [Source:HGNC Symbol;Acc:HGNC:10593]	0.66	1.53E-03
NRP1	neuropilin 1 [Source:HGNC Symbol;Acc:HGNC:8004]	0.65	1.39E-02
NCOA3	nuclear receptor coactivator 3 [Source:HGNC Symbol;Acc:HGNC:7670]	0.65	2.54E-02
SLC20A2	solute carrier family 20 member 2 [Source:HGNC Symbol;Acc:HGNC:10947]	0.65	7.79E-03
PIFO	primary cilia formation [Source:HGNC Symbol;Acc:HGNC:27009]	0.64	1.33E-02
CTNNB1	catenin beta 1 [Source:HGNC Symbol;Acc:HGNC:2514]	0.64	4.68E-02
ANP32A	acidic nuclear phosphoprotein 32 family member A [Source:HGNC Symbol;Acc:HGNC:13233]	0.64	3.81E-02
GPD1L	glycerol-3-phosphate dehydrogenase 1 like [Source:HGNC Symbol;Acc:HGNC:28956]	0.62	3.62E-02
PDCD11	programmed cell death 11 [Source:HGNC Symbol;Acc:HGNC:13408]	0.62	2.76E-02
MCL1	MCL1 apoptosis regulator, BCL2 family member [Source:HGNC Symbol;Acc:HGNC:6943]	0.62	1.57E-02
LZTS3	leucine zipper tumor suppressor family member 3 [Source:HGNC Symbol;Acc:HGNC:30139]	0.62	3.85E-02
GLUL	glutamate-ammonia ligase [Source:HGNC Symbol;Acc:HGNC:4341]	0.62	1.23E-03
MFGE8	milk fat globule-EGF factor 8 protein [Source:HGNC Symbol;Acc:HGNC:7036]	0.62	2.76E-02
DOCK7	dedicator of cytokinesis 7 [Source:HGNC Symbol;Acc:HGNC:19190]	0.61	2.21E-02
SMARCC1	SWI/SNF related, matrix associated, actin dependent regulator of chromatin subfamily c member 1 [Source:HGNC Symbol;Acc:HGNC:11104]	0.61	1.33E-02
PWWP3B	PWWP domain containing 3B [Source:HGNC Symbol;Acc:HGNC:26583]	0.61	4.80E-02
DARS1	aspartyl-tRNA synthetase 1 [Source:HGNC Symbol;Acc:HGNC:2678]	0.61	3.62E-02
AC245297.1	phosphodiesterase 4D interacting protein (myomegalin) (PDE4DIP) pseudogene	0.60	2.63E-02
PARP4	poly(ADP-ribose) polymerase family member 4 [Source:HGNC Symbol;Acc:HGNC:271]	0.60	3.65E-02
TTC28	tetratricopeptide repeat domain 28 [Source:HGNC Symbol;Acc:HGNC:29179]	0.60	3.41E-02
ACADSB	acyl-CoA dehydrogenase short/branched chain [Source:HGNC Symbol;Acc:HGNC:91]	0.59	1.16E-02
MASP1	mannan binding lectin serine peptidase 1 [Source:HGNC Symbol;Acc:HGNC:6901]	0.58	4.44E-02
SET	SET nuclear proto-oncogene [Source:HGNC Symbol;Acc:HGNC:10760]	0.58	4.47E-02
DTNA	dystrobrevin alpha [Source:HGNC Symbol;Acc:HGNC:3057]	0.58	1.39E-02
ID2	inhibitor of DNA binding 2 [Source:HGNC Symbol;Acc:HGNC:5361]	0.58	1.81E-02
CBLB	Cbl proto-oncogene B [Source:HGNC Symbol;Acc:HGNC:1542]	0.56	4.26E-02
AKAP13	A-kinase anchoring protein 13 [Source:HGNC Symbol;Acc:HGNC:371]	0.55	4.48E-02
XPR1	xenotropic and polytropic retrovirus receptor 1 [Source:HGNC Symbol;Acc:HGNC:12827]	0.54	3.29E-02
GPR162	G protein-coupled receptor 162 [Source:HGNC Symbol;Acc:HGNC:16693]	0.53	4.18E-02
H3-3B	H3.3 histone B [Source:HGNC Symbol;Acc:HGNC:4765]	0.52	4.17E-02

NDRG2	NDRG family member 2 [Source:HGNC Symbol;Acc:HGNC:14460]	0.51	2.81E-02

Supplementary Table 2. 199 downregulated DEGs in the ISS μG condition compared with the ISS 1G condition.

Gene	Description	log_2(Fold change)	adjusted p-value
SLCO4C1	solute carrier organic anion transporter family member 4C1 [Source:HGNC Symbol;Acc:HGNC:23612]	-7.93	2.2E-06
HSD3B2	hydroxy-delta-5-steroid dehydrogenase, 3 beta- and steroid delta-isomerase 2 [Source:HGNC Symbol;Acc:HGNC:5218]	-7.46	7.7E-04
LINC02201	long intergenic non-protein coding RNA 2201 [Source:HGNC Symbol;Acc:HGNC:53067]	-6.42	1.1E-02
UGT8	UDP glycosyltransferase 8 [Source:HGNC Symbol;Acc:HGNC:12555]	-6.36	7.5E-03
FAM131B	family with sequence similarity 131 member B [Source:HGNC Symbol;Acc:HGNC:22202]	-6.35	6.6E-03
DNAH2	dynein axonemal heavy chain 2 [Source:HGNC Symbol;Acc:HGNC:2948]	-6.20	8.2E-04
MUC4	mucin 4, cell surface associated [Source:HGNC Symbol;Acc:HGNC:7514]	-6.08	1.4E-02
PCSK9	proprotein convertase subtilisin/kexin type 9 [Source:HGNC Symbol;Acc:HGNC:20001]	-6.06	2.5E-02
ITIH5	inter-alpha-trypsin inhibitor heavy chain 5 [Source:HGNC Symbol;Acc:HGNC:21449]	-6.01	2.9E-02
VIPR1	vasoactive intestinal peptide receptor 1 [Source:HGNC Symbol;Acc:HGNC:12694]	-6.00	1.8E-02
COL4A4	collagen type IV alpha 4 chain [Source:HGNC Symbol;Acc:HGNC:2206]	-5.73	1.2E-02
SATB2	SATB homeobox 2 [Source:HGNC Symbol;Acc:HGNC:21637]	-5.37	3.2E-02
DNAH12	dynein axonemal heavy chain 12 [Source:HGNC Symbol;Acc:HGNC:2943]	-5.18	3.7E-02
ARHGEF15	Rho guanine nucleotide exchange factor 15 [Source:HGNC Symbol;Acc:HGNC:15590]	-5.13	3.8E-02
GABRP	gamma-aminobutyric acid type A receptor subunit pi [Source:HGNC Symbol;Acc:HGNC:4089]	-5.10	8.2E-05
C5orf46	chromosome 5 open reading frame 46 [Source:HGNC Symbol;Acc:HGNC:33768]	-4.05	2.1E-02
PTPRZ1	protein tyrosine phosphatase receptor type Z1 [Source:HGNC Symbol;Acc:HGNC:9685]	-3.90	1.7E-02
EDN1	endothelin 1 [Source:HGNC Symbol;Acc:HGNC:3176]	-3.52	4.4E-02
GRAMD1C	GRAM domain containing 1C [Source:HGNC Symbol;Acc:HGNC:25252]	-3.34	2.2E-02
TRPC4	transient receptor potential cation channel subfamily C member 4 [Source:HGNC Symbol;Acc:HGNC:12336]	-3.24	1.1E-03
Z97832.2	novel transcript, antisense to SCUBE3	-3.19	3.4E-02
CDHR1	cadherin related family member 1 [Source:HGNC Symbol;Acc:HGNC:14550]	-2.98	9.8E-03
SLC38A5	solute carrier family 38 member 5 [Source:HGNC Symbol;Acc:HGNC:18070]	-2.90	4.5E-02
MMP24	matrix metallopeptidase 24 [Source:HGNC Symbol;Acc:HGNC:7172]	-2.79	6.9E-10
C1R	complement C1r [Source:HGNC Symbol;Acc:HGNC:1246]	-2.74	4.1E-09
RGCC	regulator of cell cycle [Source:HGNC Symbol;Acc:HGNC:20369]	-2.68	4.8E-03
ITGA11	integrin subunit alpha 11 [Source:HGNC Symbol;Acc:HGNC:6136]	-2.65	1.4E-04
MFSD2A	major facilitator superfamily domain containing 2A [Source:HGNC Symbol;Acc:HGNC:25897]	-2.59	3.3E-03

NECAB2	N-terminal EF-hand calcium binding protein 2 [Source:HGNC Symbol;Acc:HGNC:23746]	-2.55	3.9E-04
MYOT	myotilin [Source:HGNC Symbol;Acc:HGNC:12399]	-2.36	3.4E-02
PROCR	protein C receptor [Source:HGNC Symbol;Acc:HGNC:9452]	-2.33	2.6E-02
COL26A1	collagen type XXVI alpha 1 chain [Source:HGNC Symbol;Acc:HGNC:18038]	-2.28	9.7E-04
NTRK1	neurotrophic receptor tyrosine kinase 1 [Source:HGNC Symbol;Acc:HGNC:8031]	-2.27	1.2E-03
HPX	hemopexin [Source:HGNC Symbol;Acc:HGNC:5171]	-2.23	3.4E-02
PHF24	PHD finger protein 24 [Source:HGNC Symbol;Acc:HGNC:29180]	-2.21	9.4E-04
AC018647.1	novel transcript	-2.13	1.1E-03
SFRP5	secreted frizzled related protein 5 [Source:HGNC Symbol;Acc:HGNC:10779]	-2.03	4.4E-11
ENC1	ectodermal-neural cortex 1 [Source:HGNC Symbol;Acc:HGNC:3345]	-2.00	8.3E-03
AC023481.1	novel transcript	-1.96	3.3E-02
SLITRK4	SLIT and NTRK like family member 4 [Source:HGNC Symbol;Acc:HGNC:23502]	-1.94	4.8E-02
NIPAL4	NIPA like domain containing 4 [Source:HGNC Symbol;Acc:HGNC:28018]	-1.87	1.1E-04
ANXA1	annexin A1 [Source:HGNC Symbol;Acc:HGNC:533]	-1.83	4.5E-05
LIMA1	LIM domain and actin binding 1 [Source:HGNC Symbol;Acc:HGNC:24636]	-1.79	1.1E-02
HPSE2	heparanase 2 (inactive) [Source:HGNC Symbol;Acc:HGNC:18374]	-1.76	1.2E-02
GPC4	glypican 4 [Source:HGNC Symbol;Acc:HGNC:4452]	-1.74	8.7E-06
INHBA	inhibin subunit beta A [Source:HGNC Symbol;Acc:HGNC:6066]	-1.71	1.7E-02
ERICH5	glutamate rich 5 [Source:HGNC Symbol;Acc:HGNC:26823]	-1.70	5.7E-03
SLC35F2	solute carrier family 35 member F2 [Source:HGNC Symbol;Acc:HGNC:23615]	-1.68	3.1E-03
AC109583.1	Probable threonine protease PRSS50 [Source:UniProtKB/Swiss-Prot;Acc:Q9UI38]	-1.65	4.0E-02
SNCAIP	synuclein alpha interacting protein [Source:HGNC Symbol;Acc:HGNC:11139]	-1.64	2.4E-03
CDC42EP2	CDC42 effector protein 2 [Source:HGNC Symbol;Acc:HGNC:16263]	-1.63	7.3E-03
RASGRP1	RAS guanyl releasing protein 1 [Source:HGNC Symbol;Acc:HGNC:9878]	-1.56	2.0E-03
MYRF	myelin regulatory factor [Source:HGNC Symbol;Acc:HGNC:1181]	-1.55	8.9E-03
CD24	CD24 molecule [Source:HGNC Symbol;Acc:HGNC:1645]	-1.54	6.2E-14
GPR176	G protein-coupled receptor 176 [Source:HGNC Symbol;Acc:HGNC:32370]	-1.48	2.5E-03
ELMO1	engulfment and cell motility 1 [Source:HGNC Symbol;Acc:HGNC:16286]	-1.47	5.5E-03
CTSV	cathepsin V [Source:HGNC Symbol;Acc:HGNC:2538]	-1.44	6.3E-06
TCIRG1	T cell immune regulator 1, ATPase H+ transporting V0 subunit a3 [Source:HGNC Symbol;Acc:HGNC:11647]	-1.43	4.9E-03
LDB2	LIM domain binding 2 [Source:HGNC Symbol;Acc:HGNC:6533]	-1.42	9.1E-04
TAGLN	transgelin [Source:HGNC Symbol;Acc:HGNC:11553]	-1.40	3.7E-05
EFHD1	EF-hand domain family member D1 [Source:HGNC Symbol;Acc:HGNC:29556]	-1.37	3.8E-02
PPM1H	protein phosphatase, Mg2+/Mn2+ dependent 1H [Source:HGNC Symbol;Acc:HGNC:18583]	-1.36	1.9E-02
ACAT2	acetyl-CoA acetyltransferase 2 [Source:HGNC Symbol;Acc:HGNC:94]	-1.36	2.3E-03

P3H3	prolyl 3-hydroxylase 3 [Source:HGNC Symbol;Acc:HGNC:19318]	-1.36	2.1E-02
NDNF	neuron derived neurotrophic factor [Source:HGNC Symbol;Acc:HGNC:26256]	-1.35	1.4E-02
LAMC2	laminin subunit gamma 2 [Source:HGNC Symbol;Acc:HGNC:6493]	-1.33	2.3E-02
MATN2	matrilin 2 [Source:HGNC Symbol;Acc:HGNC:6908]	-1.32	2.3E-09
PLPPR4	phospholipid phosphatase related 4 [Source:HGNC Symbol;Acc:HGNC:23496]	-1.31	8.3E-03
HCN3	hyperpolarization activated cyclic nucleotide gated potassium channel 3 [Source:HGNC Symbol;Acc:HGNC:19183]	-1.29	8.6E-03
ACTA1	actin alpha 1, skeletal muscle [Source:HGNC Symbol;Acc:HGNC:129]	-1.29	9.1E-04
RELB	RELB proto-oncogene, NF-kB subunit [Source:HGNC Symbol;Acc:HGNC:9956]	-1.28	1.1E-02
KMT2D	lysine methyltransferase 2D [Source:HGNC Symbol;Acc:HGNC:7133]	-1.27	1.0E-02
CRABP2	cellular retinoic acid binding protein 2 [Source:HGNC Symbol;Acc:HGNC:2339]	-1.26	1.8E-09
DDIT3	DNA damage inducible transcript 3 [Source:HGNC Symbol;Acc:HGNC:2726]	-1.23	1.7E-02
MT-ND6	mitochondrially encoded NADH:ubiquinone oxidoreductase core subunit 6 [Source:HGNC Symbol;Acc:HGNC:7462]	-1.23	1.5E-04
ARRB1	arrestin beta 1 [Source:HGNC Symbol;Acc:HGNC:711]	-1.23	1.9E-02
RNF19B	ring finger protein 19B [Source:HGNC Symbol;Acc:HGNC:26886]	-1.23	5.9E-03
RAI14	retinoic acid induced 14 [Source:HGNC Symbol;Acc:HGNC:14873]	-1.22	1.3E-04
TKT	transketolase [Source:HGNC Symbol;Acc:HGNC:11834]	-1.21	1.9E-04
EHD2	EH domain containing 2 [Source:HGNC Symbol;Acc:HGNC:3243]	-1.20	2.2E-02
SEC31B	SEC31 homolog B, COPII coat complex component [Source:HGNC Symbol;Acc:HGNC:23197]	-1.17	7.5E-03
ASXL3	ASXL transcriptional regulator 3 [Source:HGNC Symbol;Acc:HGNC:29357]	-1.16	1.9E-02
GNA11	G protein subunit alpha 11 [Source:HGNC Symbol;Acc:HGNC:4379]	-1.16	8.9E-03
MYO1D	myosin ID [Source:HGNC Symbol;Acc:HGNC:7598]	-1.15	2.3E-03
MGST1	microsomal glutathione S-transferase 1 [Source:HGNC Symbol;Acc:HGNC:7061]	-1.12	4.6E-04
THBS1	thrombospondin 1 [Source:HGNC Symbol;Acc:HGNC:11785]	-1.12	7.7E-03
BRD1	bromodomain containing 1 [Source:HGNC Symbol;Acc:HGNC:1102]	-1.12	3.3E-02
CMTM5	CKLF like MARVEL transmembrane domain containing 5 [Source:HGNC Symbol;Acc:HGNC:19176]	-1.11	1.3E-02
TP53I3	tumor protein p53 inducible protein 3 [Source:HGNC Symbol;Acc:HGNC:19373]	-1.11	3.0E-03
CORO7	coronin 7 [Source:HGNC Symbol;Acc:HGNC:26161]	-1.10	1.2E-02
S100A10	S100 calcium binding protein A10 [Source:HGNC Symbol;Acc:HGNC:10487]	-1.09	7.9E-03
ACTB	actin beta [Source:HGNC Symbol;Acc:HGNC:132]	-1.09	5.0E-03
H2BC4	H2B clustered histone 4 [Source:HGNC Symbol;Acc:HGNC:4757]	-1.07	3.8E-02
IDI1	isopentenyl-diphosphate delta isomerase 1 [Source:HGNC Symbol;Acc:HGNC:5387]	-1.06	1.3E-02
VAMP1	vesicle associated membrane protein 1 [Source:HGNC Symbol;Acc:HGNC:12642]	-1.06	1.5E-02
CAPN5	calpain 5 [Source:HGNC Symbol;Acc:HGNC:1482]	-1.05	4.2E-03

TRIO	trio Rho guanine nucleotide exchange factor [Source:HGNC Symbol;Acc:HGNC:12303]	-1.05	7.3E-03
COL1A1	collagen type I alpha 1 chain [Source:HGNC Symbol;Acc:HGNC:2197]	-1.05	2.6E-02
COTL1	coactosin like F-actin binding protein 1 [Source:HGNC Symbol;Acc:HGNC:18304]	-1.04	4.9E-03
TMEM97	transmembrane protein 97 [Source:HGNC Symbol;Acc:HGNC:28106]	-1.03	3.4E-02
CLK1	CDC like kinase 1 [Source:HGNC Symbol;Acc:HGNC:2068]	-1.02	1.1E-03
ADA	adenosine deaminase [Source:HGNC Symbol;Acc:HGNC:186]	-1.02	1.2E-02
INHA	inhibin subunit alpha [Source:HGNC Symbol;Acc:HGNC:6065]	-1.01	4.7E-02
LRRN3	leucine rich repeat neuronal 3 [Source:HGNC Symbol;Acc:HGNC:17200]	-1.01	2.8E-02
NAGLU	N-acetyl-alpha-glucosaminidase [Source:HGNC Symbol;Acc:HGNC:7632]	-1.00	5.0E-04
TCN2	transcobalamin 2 [Source:HGNC Symbol;Acc:HGNC:11653]	-0.99	1.5E-02
EHD3	EH domain containing 3 [Source:HGNC Symbol;Acc:HGNC:3244]	-0.99	1.1E-03
TXNL4B	thioredoxin like 4B [Source:HGNC Symbol;Acc:HGNC:26041]	-0.98	3.9E-02
MSMO1	methylsterol monooxygenase 1 [Source:HGNC Symbol;Acc:HGNC:10545]	-0.98	1.3E-02
CLK4	CDC like kinase 4 [Source:HGNC Symbol;Acc:HGNC:13659]	-0.98	5.2E-04
PRSS23	serine protease 23 [Source:HGNC Symbol;Acc:HGNC:14370]	-0.98	5.9E-04
PAOX	polyamine oxidase [Source:HGNC Symbol;Acc:HGNC:20837]	-0.98	1.8E-02
EMP2	epithelial membrane protein 2 [Source:HGNC Symbol;Acc:HGNC:3334]	-0.97	2.2E-04
CEP164	centrosomal protein 164 [Source:HGNC Symbol;Acc:HGNC:29182]	-0.96	3.8E-02
ZNF467	zinc finger protein 467 [Source:HGNC Symbol;Acc:HGNC:23154]	-0.95	1.4E-02
CALD1	caldesmon 1 [Source:HGNC Symbol;Acc:HGNC:1441]	-0.95	3.6E-02
ACHE	acetylcholinesterase (Cartwright blood group) [Source:HGNC Symbol;Acc:HGNC:108]	-0.95	1.7E-02
LIMK2	LIM domain kinase 2 [Source:HGNC Symbol;Acc:HGNC:6614]	-0.94	1.2E-02
FAM189A2	family with sequence similarity 189 member A2 [Source:HGNC Symbol;Acc:HGNC:24820]	-0.94	9.4E-03
USP7	ubiquitin specific peptidase 7 [Source:HGNC Symbol;Acc:HGNC:12630]	-0.94	1.1E-02
GBP2	guanylate binding protein 2 [Source:HGNC Symbol;Acc:HGNC:4183]	-0.93	1.7E-02
MFAP4	microfibril associated protein 4 [Source:HGNC Symbol;Acc:HGNC:7035]	-0.93	3.3E-02
IQGAP1	IQ motif containing GTPase activating protein 1 [Source:HGNC Symbol;Acc:HGNC:6110]	-0.92	3.7E-02
FAM89A	family with sequence similarity 89 member A [Source:HGNC Symbol;Acc:HGNC:25057]	-0.92	1.4E-02
NAGPA	N-acetylglucosamine-1-phosphodiester alpha-N-acetylglucosaminidase [Source:HGNC Symbol;Acc:HGNC:17378]	-0.90	2.1E-02
RHPN1	rhophilin Rho GTPase binding protein 1 [Source:HGNC Symbol;Acc:HGNC:19973]	-0.90	1.5E-02
PLS3	plastin 3 [Source:HGNC Symbol;Acc:HGNC:9091]	-0.90	1.8E-04
SGK1	serum/glucocorticoid regulated kinase 1 [Source:HGNC Symbol;Acc:HGNC:10810]	-0.90	4.4E-02
GLRX	glutaredoxin [Source:HGNC Symbol;Acc:HGNC:4330]	-0.90	3.0E-02
GSDME	gasdermin E [Source:HGNC Symbol;Acc:HGNC:2810]	-0.89	4.7E-03
SQLE	squalene epoxidase [Source:HGNC Symbol;Acc:HGNC:11279]	-0.89	1.0E-02

ITGB5	integrin subunit beta 5 [Source:HGNC Symbol;Acc:HGNC:6160]	-0.89	3.6E-02
P3H1	prolyl 3-hydroxylase 1 [Source:HGNC Symbol;Acc:HGNC:19316]	-0.89	2.0E-02
DAB2	DAB adaptor protein 2 [Source:HGNC Symbol;Acc:HGNC:2662]	-0.88	3.4E-02
ASS1	argininosuccinate synthase 1 [Source:HGNC Symbol;Acc:HGNC:758]	-0.88	5.5E-03
CSRP2	cysteine and glycine rich protein 2 [Source:HGNC Symbol;Acc:HGNC:2470]	-0.88	4.9E-03
PLXNA1	plexin A1 [Source:HGNC Symbol;Acc:HGNC:9099]	-0.88	4.7E-02
ARSA	arylsulfatase A [Source:HGNC Symbol;Acc:HGNC:713]	-0.88	1.0E-02
FHL1	four and a half LIM domains 1 [Source:HGNC Symbol;Acc:HGNC:3702]	-0.87	2.3E-02
HSD17B14	hydroxysteroid 17-beta dehydrogenase 14 [Source:HGNC Symbol;Acc:HGNC:23238]	-0.86	4.2E-02
MAFG	MAF bZIP transcription factor G [Source:HGNC Symbol;Acc:HGNC:6781]	-0.86	9.9E-03
EFNB2	ephrin B2 [Source:HGNC Symbol;Acc:HGNC:3227]	-0.84	2.3E-03
TPM2	tropomyosin 2 [Source:HGNC Symbol;Acc:HGNC:12011]	-0.84	4.1E-04
VAT1L	vesicle amine transport 1 like [Source:HGNC Symbol;Acc:HGNC:29315]	-0.84	5.8E-03
GRN	granulin precursor [Source:HGNC Symbol;Acc:HGNC:4601]	-0.84	7.3E-03
C3orf70	chromosome 3 open reading frame 70 [Source:HGNC Symbol;Acc:HGNC:33731]	-0.83	3.7E-02
FBXL6	F-box and leucine rich repeat protein 6 [Source:HGNC Symbol;Acc:HGNC:13603]	-0.83	2.1E-02
PLAAT3	phospholipase A and acyltransferase 3 [Source:HGNC Symbol;Acc:HGNC:17825]	-0.83	1.4E-02
GUCY1A1	guanylate cyclase 1 soluble subunit alpha 1 [Source:HGNC Symbol;Acc:HGNC:4685]	-0.83	2.5E-02
TENT5A	terminal nucleotidyltransferase 5A [Source:HGNC Symbol;Acc:HGNC:18345]	-0.81	3.7E-03
P2RY14	purinergic receptor P2Y14 [Source:HGNC Symbol;Acc:HGNC:16442]	-0.80	1.3E-02
HTRA1	HtrA serine peptidase 1 [Source:HGNC Symbol;Acc:HGNC:9476]	-0.79	3.8E-02
PAXIP1-AS1	PAXIP1 antisense RNA 1 (head to head) [Source:HGNC Symbol;Acc:HGNC:27328]	-0.79	3.4E-02
LGR4	leucine rich repeat containing G protein-coupled receptor 4 [Source:HGNC Symbol;Acc:HGNC:13299]	-0.78	4.2E-02
SRPX	sushi repeat containing protein X-linked [Source:HGNC Symbol;Acc:HGNC:11309]	-0.78	3.3E-02
MVD	mevalonate diphosphate decarboxylase [Source:HGNC Symbol;Acc:HGNC:7529]	-0.77	3.3E-02
PUM3	pumilio RNA binding family member 3 [Source:HGNC Symbol;Acc:HGNC:29676]	-0.76	4.1E-02
TENM4	teneurin transmembrane protein 4 [Source:HGNC Symbol;Acc:HGNC:29945]	-0.76	1.7E-02
ULK3	unc-51 like kinase 3 [Source:HGNC Symbol;Acc:HGNC:19703]	-0.75	1.5E-03
MOV10	Mov10 RISC complex RNA helicase [Source:HGNC Symbol;Acc:HGNC:7200]	-0.74	4.9E-03
F11R	F11 receptor [Source:HGNC Symbol;Acc:HGNC:14685]	-0.73	5.1E-03
RSRP1	arginine and serine rich protein 1 [Source:HGNC Symbol;Acc:HGNC:25234]	-0.73	1.1E-02
CXXC5	CXXC finger protein 5 [Source:HGNC Symbol;Acc:HGNC:26943]	-0.73	2.4E-02
LRRC8D	leucine rich repeat containing 8 VRAC subunit D [Source:HGNC Symbol;Acc:HGNC:16992]	-0.72	2.7E-02

CERCAM	cerebral endothelial cell adhesion molecule [Source:HGNC Symbol;Acc:HGNC:23723]	-0.72	4.9E-03
SUMF1	sulfatase modifying factor 1 [Source:HGNC Symbol;Acc:HGNC:20376]	-0.72	9.8E-03
MVK	mevalonate kinase [Source:HGNC Symbol;Acc:HGNC:7530]	-0.72	1.8E-02
SWAP70	switching B cell complex subunit SWAP70 [Source:HGNC Symbol;Acc:HGNC:17070]	-0.71	3.8E-02
SLIT2	slit guidance ligand 2 [Source:HGNC Symbol;Acc:HGNC:11086]	-0.69	1.4E-02
S100A11	S100 calcium binding protein A11 [Source:HGNC Symbol;Acc:HGNC:10488]	-0.68	1.3E-02
FGFR2	fibroblast growth factor receptor 2 [Source:HGNC Symbol;Acc:HGNC:3689]	-0.68	4.4E-03
SPARC	secreted protein acidic and cysteine rich [Source:HGNC Symbol;Acc:HGNC:11219]	-0.68	1.7E-02
MYO5B	myosin VB [Source:HGNC Symbol;Acc:HGNC:7603]	-0.67	3.7E-02
HEXA	hexosaminidase subunit alpha [Source:HGNC Symbol;Acc:HGNC:4878]	-0.67	3.4E-02
PRDX1	peroxiredoxin 1 [Source:HGNC Symbol;Acc:HGNC:9352]	-0.67	1.8E-04
F2R	coagulation factor II thrombin receptor [Source:HGNC Symbol;Acc:HGNC:3537]	-0.66	2.6E-02
ACTN1	actinin alpha 1 [Source:HGNC Symbol;Acc:HGNC:163]	-0.66	1.2E-03
GIPC1	GIPC PDZ domain containing family member 1 [Source:HGNC Symbol;Acc:HGNC:1226]	-0.65	4.5E-03
MACROH2A1	macroH2A.1 histone [Source:HGNC Symbol;Acc:HGNC:4740]	-0.64	2.6E-02
KIF13A	kinesin family member 13A [Source:HGNC Symbol;Acc:HGNC:14566]	-0.64	2.8E-02
TP53I11	tumor protein p53 inducible protein 11 [Source:HGNC Symbol;Acc:HGNC:16842]	-0.63	1.5E-03
PRCP	prolylcarboxypeptidase [Source:HGNC Symbol;Acc:HGNC:9344]	-0.62	3.6E-02
TULP3	TUB like protein 3 [Source:HGNC Symbol;Acc:HGNC:12425]	-0.62	2.8E-02
CNN1	calponin 1 [Source:HGNC Symbol;Acc:HGNC:2155]	-0.61	4.0E-02
CERS5	ceramide synthase 5 [Source:HGNC Symbol;Acc:HGNC:23749]	-0.60	9.4E-03
ANXA2	annexin A2 [Source:HGNC Symbol;Acc:HGNC:537]	-0.60	6.7E-03
SERPINI1	serpin family I member 1 [Source:HGNC Symbol;Acc:HGNC:8943]	-0.60	3.3E-02
ANO10	anoctamin 10 [Source:HGNC Symbol;Acc:HGNC:25519]	-0.59	2.8E-02
EPHB2	EPH receptor B2 [Source:HGNC Symbol;Acc:HGNC:3393]	-0.59	9.1E-03
FLNA	filamin A [Source:HGNC Symbol;Acc:HGNC:3754]	-0.58	2.0E-02
AKR1B1	aldo-keto reductase family 1 member B [Source:HGNC Symbol;Acc:HGNC:381]	-0.58	2.4E-02
ACTA2	actin alpha 2, smooth muscle [Source:HGNC Symbol;Acc:HGNC:130]	-0.56	3.8E-02
PRKRIP1	PRKR interacting protein 1 [Source:HGNC Symbol;Acc:HGNC:21894]	-0.56	2.5E-02
TRIM54	tripartite motif containing 54 [Source:HGNC Symbol;Acc:HGNC:16008]	-0.56	2.8E-02
NFE2L2	nuclear factor, erythroid 2 like 2 [Source:HGNC Symbol;Acc:HGNC:7782]	-0.53	4.9E-02
RCN1	reticulocalbin 1 [Source:HGNC Symbol;Acc:HGNC:9934]	-0.53	5.0E-02
DDX28	DEAD-box helicase 28 [Source:HGNC Symbol;Acc:HGNC:17330]	-0.52	3.5E-02
ABHD4	abhydrolase domain containing 4 [Source:HGNC Symbol;Acc:HGNC:20154]	-0.50	3.3E-02
TSC22D3	TSC22 domain family member 3 [Source:HGNC Symbol;Acc:HGNC:3051]	-0.45	3.6E-02

Supplementary Table 3. Select upregulated GO terms in the ISS μG condition compared with the ISS 1G condition.

Ontology	ID	Description	adjusted p-value	Count
BP	GO:0003012	muscle system process	4.91E-09	29
BP	GO:0006936	muscle contraction	7.77E-09	25
BP	GO:0060537	muscle tissue development	7.19E-07	24
BP	GO:0014706	striated muscle tissue development	5.92E-06	22
BP	GO:0006941	striated muscle contraction	9.27E-06	15
BP	GO:0006942	regulation of striated muscle contraction	2.55E-05	11
BP	GO:0050673	epithelial cell proliferation	3.41E-05	22
BP	GO:0003015	heart process	8.21E-05	16
BP	GO:0090257	regulation of muscle system process	8.21E-05	16
BP	GO:0048738	cardiac muscle tissue development	1.54E-04	15
BP	GO:0003229	ventricular cardiac muscle tissue development	1.54E-04	8
BP	GO:0050679	positive regulation of epithelial cell proliferation	1.54E-04	14
BP	GO:0051302	regulation of cell division	1.54E-04	13
BP	GO:0050678	regulation of epithelial cell proliferation	1.54E-04	19
BP	GO:0060047	heart contraction	1.54E-04	15
BP	GO:0003205	cardiac chamber development	2.90E-04	12
BP	GO:1903522	regulation of blood circulation	2.90E-04	15
BP	GO:0006937	regulation of muscle contraction	4.44E-04	12
BP	GO:0008016	regulation of heart contraction	5.91E-04	13
BP	GO:0003231	cardiac ventricle development	8.53E-04	10
MF	GO:0140416	transcription regulator inhibitor activity	2.94E-03	5
MF	GO:0044325	transmembrane transporter binding	2.94E-03	10
MF	GO:0034237	protein kinase A regulatory subunit binding	2.94E-03	5
MF	GO:0051018	protein kinase A binding	7.32E-03	6
MF	GO:0140678	molecular function inhibitor activity	3.26E-02	8
MF	GO:0017154	semaphorin receptor activity	4.03E-02	3
CC	GO:0030016	myofibril	1.76E-03	13
CC	GO:0043292	contractile fiber	1.76E-03	13
CC	GO:0044853	plasma membrane raft	1.76E-03	9
CC	GO:0030017	sarcomere	1.76E-03	12
CC	GO:0045121	membrane raft	1.86E-03	15
CC	GO:0098857	membrane microdomain	1.86E-03	15

Ontology	ID	Description	adjusted p-value	Count
CC	GO:0014704	intercalated disc	2.19E-03	6
CC	GO:0042383	sarcolemma	3.43E-03	9
CC	GO:0051233	spindle midzone	3.72E-03	5
CC	GO:0005901	caveola	3.79E-03	7
CC	GO:0030018	Z disc	9.17E-03	8
CC	GO:0002116	semaphorin receptor complex	9.17E-03	3
CC	GO:0044291	cell-cell contact zone	9.17E-03	6
CC	GO:0034703	cation channel complex	1.04E-02	10
CC	GO:0019897	extrinsic component of plasma membrane	1.04E-02	9
CC	GO:0031674	I band	1.05E-02	8

Supplementary Table 4. DEGs associated with regulation of cell division.

**Gene**	**Description**	**log_2(fold change)**	**adjusted p-value**
KIF14	kinesin family member 14	2.11	2.42E-03
IGF2	insulin like growth factor 2	2.07	9.89E-03
KIF18B	kinesin family member 18B	1.89	3.23E-02
SUSD2	sushi domain containing 2	1.88	2.86E-08
RBL1	RB transcriptional corepressor like 1	1.32	1.19E-02
FGF1	fibroblast growth factor 1	1.3	5.54E-07
AURKB	aurora kinase B	1.28	2.47E-02
LBH	LBH regulator of WNT signaling pathway	0.97	7.71E-03
PDGFD	platelet derived growth factor D	0.93	1.28E-02
CDC42	cell division cycle 42	0.9	9.44E-04
THBS4	thrombospondin 4	0.9	5.96E-03
TXNIP	thioredoxin interacting protein	0.85	1.55E-02
NCOA3	nuclear receptor coactivator 3	0.65	2.54E-02

Supplementary Table 5. DEGs associated with muscle cell differentiation.

**Gene**	**Description**	**log_2(fold change)**	**adjusted p-value**
MYL2	myosin light chain 2	2.44	4.39E-03
IGFBP5	insulin like growth factor binding protein 5	2.19	6.94E-10
IGF2	IL4R (interleukin 4 receptor	1.86	6.31E-07
EDNRB	endothelin receptor type B	1.15	6.90E-03
NFATC2	nuclear factor of activated T cells 2	1.08	2.67E-02
MYO18B	myosin XVIIIB	0.96	2.31E-03
LMOD2	leiomodin 2	0.82	4.36E-03
ANK2	ankyrin 2	0.79	4.68E-02
AKAP6	A-kinase anchoring protein 6	0.69	4.63E-02
CTNNB1	catenin beta 1	0.64	4.68E-02
ID2	inhibitor of DNA binding 2	0.58	1.81E-02
AKAP13	A-kinase anchoring protein 13	0.55	4.48E-02

Supplementary Table 6. Select downregulated GO terms in in the ISS µG condition compared with the ISS 1G condition.

Ontology	ID	Description	adjusted p-value	Count
BP	GO:0007015	actin filament organization	1.75E-06	22
BP	GO:0051017	actin filament bundle assembly	7.69E-05	12
BP	GO:0061572	actin filament bundle organization	7.69E-05	12
BP	GO:0048511	rhythmic process	1.36E-03	14
BP	GO:0042060	wound healing	3.17E-03	16
BP	GO:0050818	regulation of coagulation	3.43E-03	7
BP	GO:0007623	circadian rhythm	3.72E-03	11
BP	GO:0090130	tissue migration	6.22E-03	14
BP	GO:0061564	axon development	6.22E-03	16
BP	GO:0043542	endothelial cell migration	7.05E-03	12
BP	GO:0050878	regulation of body fluid levels	7.05E-03	14
BP	GO:0031032	actomyosin structure organization	7.05E-03	10
BP	GO:0010976	positive regulation of neuron projection development	8.87E-03	9
BP	GO:0010975	regulation of neuron projection development	8.87E-03	15
BP	GO:1903034	regulation of response to wounding	9.34E-03	9
BP	GO:0030193	regulation of blood coagulation	9.53E-03	6
BP	GO:0031346	positive regulation of cell projection organization	9.92E-03	13
BP	GO:1900046	regulation of hemostasis	1.00E-02	6
BP	GO:0007596	blood coagulation	1.02E-02	10
BP	GO:0007409	axonogenesis	1.02E-02	14
MF	GO:0003779	actin binding	1.34E-03	17
MF	GO:0051015	actin filament binding	3.38E-03	11
MF	GO:0045296	cadherin binding	5.84E-03	13
MF	GO:0005201	extracellular matrix structural constituent	8.06E-03	9
MF	GO:0050840	extracellular matrix binding	2.45E-02	5
MF	GO:0044548	S100 protein binding	2.73E-02	3
MF	GO:0015929	hexosaminidase activity	2.90E-02	3
MF	GO:0005518	collagen binding	3.96E-02	5
MF	GO:0098641	cadherin binding involved in cell-cell adhesion	3.96E-02	3
CC	GO:0062023	collagen-containing extracellular matrix	1.45E-06	20
CC	GO:0005884	actin filament	2.31E-04	9
CC	GO:0005925	focal adhesion	2.59E-04	16
CC	GO:0030055	cell-substrate junction	2.59E-04	16
CC	GO:0005938	cell cortex	5.33E-04	13
CC	GO:0005788	endoplasmic reticulum lumen	2.55E-03	12

Ontology	ID	Description	adjusted p-value	Count
CC	GO:0098978	glutamatergic synapse	4.54E-03	12
CC	GO:0005775	vacuolar lumen	1.06E-02	8
CC	GO:0043202	lysosomal lumen	1.17E-02	6
CC	GO:0043292	contractile fiber	1.55E-02	9
CC	GO:0030863	cortical cytoskeleton	1.70E-02	6
CC	GO:0005766	primary lysosome	1.76E-02	7
CC	GO:0042582	azurophil granule	1.76E-02	7
CC	GO:0032432	actin filament bundle	2.27E-02	5
CC	GO:0060205	cytoplasmic vesicle lumen	2.38E-02	10
CC	GO:0031983	vesicle lumen	2.38E-02	10

Supplementary Table 7. DEGs associated with cardiac contraction and conduction.

**Gene**	**Description**	**log_2(fold change)**	**adjusted p-value**
MYL2	myosin light chain 2	2.44	4.39E-03
TNNI3	troponin I3	1.64	2.58E-09
ATP2B4	ATPase plasma membrane Ca2+ transporting 4	1.2	2.52E-05
PRKACA	protein kinase cAMP-activated catalytic subunit a	0.95	4.73E-02
RYR2	ryanodine receptor 2	0.88	3.29E-03
TCAP	titin-cap	0.85	1.11E-03
ANK2	ankyrin 2	0.79	4.68E-02
SCN5A	sodium voltage-gated channel alpha subunit 5	0.66	1.53E-03

Supplementary Table 8. DEGs associated with regulation of ECMs.

**Gene**	**Description**	**log_2(fold change)**	**adjusted p-value**
ITIH5	inter-alpha-trypsin inhibitor heavy chain 5	-6.01	2.88E-02
COL4A4	collagen type IV alpha 4 chain	-5.73	1.16E-02
PTPRZ1	protein tyrosine phosphatase receptor type Z1	-3.9	1.74E-02
COL26A1	collagen type XXVI alpha 1 chain	-2.28	9.69E-04
HPX	hemopexin	-2.23	3.38E-02
ANXA1	annexin A1	-1.83	4.48E-05
GPC4	glypican 4	-1.74	8.72E-06
LAMC2	laminin subunit gamma 2	-1.33	2.26E-02
MATN2	matrilin 2	-1.32	2.28E-09
THBS1	thrombospondin 1	-1.12	7.71E-03
S100A10	S100 calcium binding protein A10	-1.09	7.92E-03
COL1A1	collagen type I alpha 1 chain	-1.05	2.57E-02
ACHE	acetylcholinesterase	-0.95	1.74E-02
MFAP4	microfibril associated protein 4	-0.93	3.32E-02
P3H1	prolyl 3-hydroxylase 1	-0.89	2.02E-02
HTRA1	HtrA serine peptidase 1	-0.79	3.81E-02
SRPX	sushi repeat containing protein X-linked	-0.78	3.29E-02
FGFR2	fibroblast growth factor receptor 2	-0.68	4.39E-03
SPARC	secreted protein acidic and cysteine rich	-0.68	1.67E-02
ANXA2	annexin A2	-0.6	6.74E-03

Supplementary Table 9. DEGs associated with focal adhesion.

**Gene**	**Description**	**log_2(fold change)**	**adjusted p-value**
ITGA11	integrin subunit alpha 11	-2.65	1.40E-04
PROCR	protein C receptor	-2.33	2.55E-02
ANXA1	annexin A1	-1.83	4.48E-05
LIMA1	LIM domain and actin binding 1	-1.79	1.11E-02
ACTB	actin beta	-1.09	5.05E-03
CAPN5	calpain 5	-1.05	4.23E-03
EHD3	EH domain containing 3	-0.99	1.06E-03
IQGAP1	IQ motif containing GTPase activating protein 1	-0.92	3.73E-02
ITGB5	integrin subunit beta 5	-0.89	3.62E-02
DAB2	DAB adaptor protein 2	-0.88	3.36E-02
CSRP2	cysteine and glycine rich protein 2	-0.88	4.93E-03
FHL1	four and a half LIM domains 1	-0.87	2.26E-02
EFNB2	ephrin B2	-0.84	2.31E-03
ACTN1	actinin alpha 1	-0.66	1.23E-03
CNN1	calponin 1	-0.61	4.01E-02

Supplementary Table 10. Common upregulated DEGs between short-term and long-term exposure to space microgravity.

**Gene**	**Description**	**log_2(fold change)**	**adjusted p-value**
ARTN	artemin [Source:HGNC Symbol;Acc:HGNC:727]	1.32	2.76E-02
PRSS35	serine protease 35 [Source:HGNC Symbol;Acc:HGNC:21387]	0.90	1.34E-03
ATP2B4	ATPase plasma membrane Ca2+ transporting 4 [Source:HGNC Symbol;Acc:HGNC:817]	0.89	1.60E-04
MYO18B	myosin XVIIIB [Source:HGNC Symbol;Acc:HGNC:18150]	0.82	2.59E-02
TFRC	transferrin receptor [Source:HGNC Symbol;Acc:HGNC:11763]	0.76	2.32E-03
CCND2	cyclin D2 [Source:HGNC Symbol;Acc:HGNC:1583]	0.67	2.34E-03
EXOC6B	exocyst complex component 6B [Source:HGNC Symbol;Acc:HGNC:17085]	0.61	1.77E-03
XPR1	xenotropic and polytropic retrovirus receptor 1 [Source:HGNC Symbol;Acc:HGNC:12827]	0.54	1.01E-02
FGFR2	fibroblast growth factor receptor 2 [Source:HGNC Symbol;Acc:HGNC:3689]	0.54	3.98E-02
CSRP2	cysteine and glycine rich protein 2 [Source:HGNC Symbol;Acc:HGNC:2470]	0.52	1.59E-02
MGST1	microsomal glutathione S-transferase 1 [Source:HGNC Symbol;Acc:HGNC:7061]	0.52	2.64E-02

Supplementary Table 11. Common downregulated DEGs between short-term and long-term exposure to space microgravity.

**Gene**	**Description**	**log_2(fold change)**	**adjusted p-value**
H2BC4	H2B clustered histone 4 [Source:HGNC Symbol;Acc:HGNC:4757]	-2.47	1.19E-02
EFEMP1	EGF containing fibulin extracellular matrix protein 1 [Source:HGNC Symbol;Acc:HGNC:3218]	-1.78	1.06E-02
MT-ND6	mitochondrially encoded NADH:ubiquinone oxidoreductase core subunit 6 [Source:HGNC Symbol;Acc:HGNC:7462]	-1.34	4.19E-02
DDIT3	DNA damage inducible transcript 3 [Source:HGNC Symbol;Acc:HGNC:2726]	-1.14	8.63E-05
ACTG1	actin gamma 1 [Source:HGNC Symbol;Acc:HGNC:144]	-0.92	1.49E-05
CDC42EP2	CDC42 effector protein 2 [Source:HGNC Symbol;Acc:HGNC:16263]	-0.84	1.28E-03
LIMA1	LIM domain and actin binding 1 [Source:HGNC Symbol;Acc:HGNC:24636]	-0.80	4.09E-02
EFNB2	ephrin B2 [Source:HGNC Symbol;Acc:HGNC:3227]	-0.73	4.23E-02
TKT	transketolase [Source:HGNC Symbol;Acc:HGNC:11834]	-0.47	3.80E-02
